# Ultrasound-Assisted Extraction of Natural Pigments From Food Processing By-Products: A Review

**DOI:** 10.3389/fnut.2022.891462

**Published:** 2022-05-24

**Authors:** Guillermo Linares, Meliza Lindsay Rojas

**Affiliations:** ^1^Departamento de Ciencias Agroindustriales, Universidad Nacional de Trujillo, Trujillo, Peru; ^2^Dirección de Investigación, Innovación y Responsabilidad Social, Universidad Privada del Norte (UPN), Trujillo, Peru

**Keywords:** emerging technology, high-power ultrasound (HPU), extraction, natural pigments, by-products

## Abstract

Ultrasound is an emerging technology, which has been highly explored in the food area to improve processes and products. When ultrasound is applied to a product with solid or fluid characteristics, the passage of acoustic waves and acoustic cavitation generates different mechanisms responsible for modifications in the original matrix of the sample. These effects of ultrasound can also be used to take advantage of by-products, for example by extracting compounds of interest, including natural pigments. Natural pigments or colorants are being highly demanded by different industries not only for color purposes but also due to their healthy properties, the greater demands in regulations and new consumer preferences. This review presents an updated critical analysis of the application of ultrasound-assisted extraction (UAE) to obtain natural pigments from food processing by-products. Initially, the ultrasound effects and mechanisms that improve the extraction of natural pigments in a fluid medium, as well as the factors that influence the extraction and the energy consumption of UAE are analyzed and described. Subsequently, the UAE application to obtain pigments belonging to the groups of carotenoids, chlorophyll, anthocyanins and betalains is evaluated. These sections detail the processing conditions, positive and negative effects, as well as possible applications of the extracted pigments. This review presents relevant information that may be useful to expand and explore new applications of ultrasound technology as well as promote the revaluation of by-products to obtain pigments that can be used in food, pharmaceutical or cosmetic industries.

## Introduction

Large amounts of by-products are generated throughout the agri-food chain, mainly from harvesting to food processing operations. By-products are the residues that can be used in future processes because they have characteristics or compounds of interest. Food by-products are characterized by having compounds such as lipids, proteins and sugars and are a potential source of bioactive compounds including natural pigments (1, 2), which can be recovered to be applied as food or non-food ingredients. According to Cano-Lamadrid and Artés-Hernández (3), among food applications, it could be mentioned the enriched minimally processed fruits, beverages and purees fortification, healthier and “clean label” bakery and confectionary products, intelligent food packaging, and edible coatings.

**FIGURE 1:**
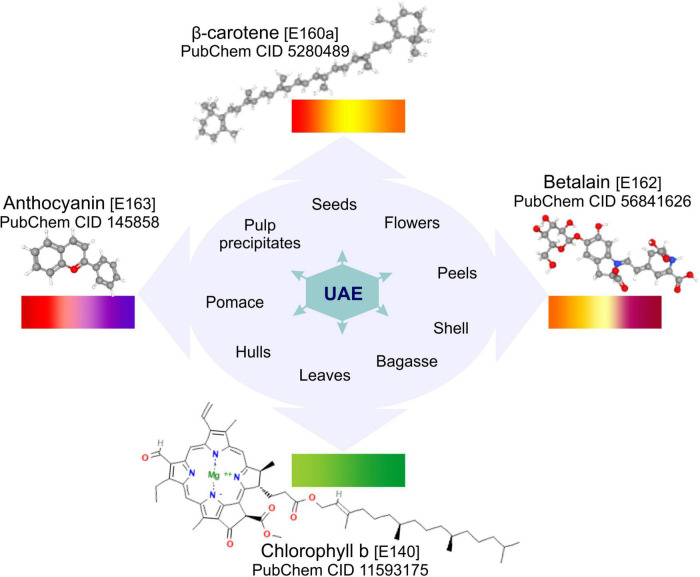
Basic molecular structure of carotenoids, anthocyanins, betalains and chlorophyll obtained from PubChem (123), the natural pigments which can be recovered by UAE from different food by-products.

Natural pigments, due to their health benefits, are increasingly preferred over synthetic pigments, the natural pigments that are extracted from by-products belong to the large groups of anthocyanins betalains, carotenoids and chlorophylls (2–4). According to Ramesh and Muthuraman (5) among the dyes of natural sources are carotenoids (for yellow, orange and deep red colors); anthocyanins (E163) (for blue, purple, red, and intermediate colors); betalains (E162) (for red-violet and yellow-orange colors) and chlorophylls (E140) (for light to deep green colors) (represented in Figure 1). Therefore, there is a growing interest in extracting pigments from natural sources, where by-products are a low-cost alternative with great potential. To recover pigments and other intracellular compounds from by-products, it is necessary to reduce the mass transfer resistances by promoting the solvent-sample contact and interactions. Different conventional and emerging technologies or methods have been applied to extract biocompounds from by-products. Among them, the conventional methods include those that have been used for a long-time such as distillation, solvent extraction, maceration, heat treatment and soxhlet extraction. However, these methods present disadvantages, they are time, energy and solvent consuming, present low extraction yields and induce the loss of thermolabile compounds. To overcome these disadvantages of conventional methods, the emerging, also called non-thermal or non-conventional, technologies are being explored. Different emerging technologies, including supercritical fluid extraction, microwave-assisted extraction, ultrasound-assisted extraction (UAE), high-pressure homogenization, pulsed electric fields, high voltage electrical discharges, light stresses, enzyme- assisted treatment, among others, have been proposed, developed, and improved. These technologies demonstrated to be a sustainable alternative to conventional extraction, showing the potential to increase the extraction yield, and decrease the extraction time, energy and solvent consumption. For more information, the recent reviews carried out by Wani et al. (2), Cano-Lamadrid and Artés-Hernández (3), Sharma et al. (4), and Carpentieri et al. (6) show information on the different conventional and emerging technologies applied to by-products to recover pigments and other compounds of interest.

Among the emerging technologies, UAE is one of the most explored and effective technology to recover natural pigments from by-products (2, 4). This technology consists of applying sound waves with specific characteristics (frequency, amplitude and wavelength) (7) to perform physicochemical changes on the propagation medium (8). Ultrasound is generated by ultrasonic transducers, these oscillatory systems turn the electric energy into acoustic waves at the desired frequency and intensity (frequency > 20 kHz, intensity > 1 W⋅cm^(–1)^) (9, 10). According to Tiwari (11) and Chemat et al. (12) the main benefits of UAE are the decrease in extraction and processing time, energy, CO emissions and solvents used, enhance extraction yield, allowing the opportunity to use aqueous extraction or alternative clean and/or green solvents by improving their extraction performance and enhance extraction of heat-sensitive components under conditions that would otherwise have low or unacceptable yields.

Although to date, there are outstanding reviews on the application of UAE in foods and natural products, or on the use of emerging technologies to produce natural pigments from by-products, there is not a recent review exclusively focused on the application of UAE to obtain natural pigments from food by-products. Therefore, this study intends to provide a critical view of the state-of-the-art regarding the application of UAE in the last 5 years to produce natural pigments from food by-products (Figure 1), focusing on ultrasound mechanisms and parameters influencing the UAE of natural pigments, the UAE processing conditions, main results and possible applications of anthocyanins, carotenoids, chlorophyll and betalains extracted from food by-products.

## Ultrasound Mechanisms, Influencing Factors and Energy Consumption in the Ultrasound-Assisted Extraction of Natural Pigments

### How Does Ultrasound Promote Pigment Extraction?

**FIGURE 2:**
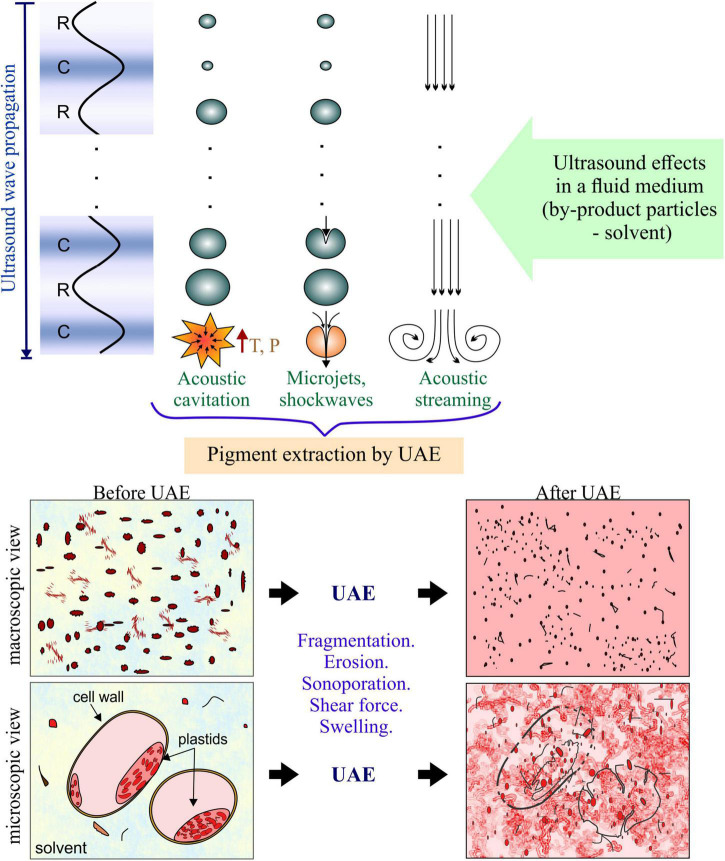
Ultrasound effects that promote mechanisms of pigment extraction through UAE processing.

The UAE is applied in a fluid medium composed of the particulate by-product (usually in powder form) dispersed in the solvent. In these fluid systems, the mechanisms of enhanced extraction by ultrasound are mainly related to hydrodynamical phenomena, which are produced by the acoustic microstreaming and acoustic cavitation (13), whose occurrence and effects on the solid-liquid interface are important and were represented in Figure 2.

When ultrasound is applied to the fluid it causes the fluid acceleration in the same direction of the wave propagation (14). This mechanism is called acoustic streaming. When the accelerated fluid rises boundaries (container walls or sample particles), it causes turbulence that increases the agitation of the bulk flow. Therefore, acoustic streaming increases the mass and heat transfer by increasing the convection (15). As the ultrasound waves are propagated in the fluid, the alternating compression (C) and expansion or rarefaction (R) cause the vaporization of the liquid generating gas bubbles, whose size is increased during R cycles and decreased during C cycles (8, 16). When the bubbles reach a specific condition, they implode dissipating a punctual (microscopic) high quantity of energy, increasing the local temperature and pressure. This implosion is called acoustic cavitation, which results in physical shearing effects (micro jetting and pressure shockwaves) (17). According to Kumar et al. (18), acoustic cavitation is the main mechanism involved in UAE.

Considering the particulate solid-solvent system during the UAE process, due to the proximity of the solid surface of particles, the cavitation bubbles often collapse asymmetrically in the surrounding liquid forming a very fast jet (with velocities in the order of 100 m⋅s^–1^) (19). This effect leads to interparticle collision, rapid heat and mass transfer at the solid surface as the boundary layer is disrupted. The accelerated interparticle collision and the implosion of cavitation bubbles on the particle surface cause particle surface erosion and fragmentation (20, 21). In addition, during acoustic cavitation, the formation of pores in the cell membranes also could occur, called “sonoporation,” which results in the release of intracellular compounds (18). In fact, pigments in plants are in organelles inside the cells, called vacuoles and plastids. In the case of anthocyanins and betalains are accumulated in the large central vacuole of most plants (5, 22), while the pigments such as chlorophyll and carotenoids are located in chloroplasts and chromoplasts plastids, respectively (23). Therefore, the rupture of these organelles and the cell wall during the UAE means a greater release of pigments.

Is important to mention, that there are other effects produced by ultrasound waves such as the microchannels formation and the “sponge effect” (24). However, these ultrasound effects have a significant impact on solid samples with a certain level of moisture (25–27). Then, considering that UAE is usually applied in a particulate fluid medium, these mechanisms are less important.

Therefore, collapsing cavitation bubbles and the propagation of the sound waves may induce different extraction mechanisms, such as fragmentation, localized erosion, pore formation, shear force, increased absorption and swelling index in the cellular matrix of the sample (18, 28). A detailed description of the extraction mechanisms induced by ultrasound is described by Chemat et al. (12). These mechanisms, in summary, result in a reduction in particle size, release of intracellular pigments, increase in the contact area of the sample with the solvent, the improvement of the solubilization of the compound, and the improvement of the transport of the solvent toward the matrix and of the compound toward the solvent (Figure 2). The increase in the extraction by UAE is attributed to the combined effect of all the mechanisms, which prevalence depends on different factors influencing the process, these factors are detailed below.

### Factors Influencing the Ultrasound-Assisted Extraction of Pigments

**FIGURE 3:**
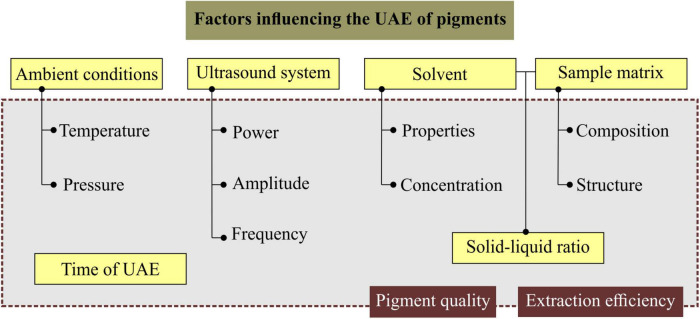
Factors and parameters that influence the UAE process and impact extraction efficiency and pigment quality.

During a UAE process, several factors can be varied or controlled depending on the objective, since their levels impact the efficiency of the process and the quality of the pigment obtained. Among the main factors and their parameters are the ambient conditions (temperature, pressure), ultrasound system (actual acoustic power, frequency and amplitude), solvent (physicochemical properties, concentration), sample matrix (composition and structure), Solid/Liquid ratio (solid = by-product sample; liquid = solvent) and time of UAE process, represented in Figure 3.

#### Temperature

Temperature is an important parameter to be controlled since it directly impacts the solute and solvent properties (such as viscosity, vapor pressure, and surface tension), which in turn impacts cavitation and its effects (12, 18). On the one hand, an increase in temperature is related to time reduction and higher extraction yield, due to increased solvent diffusivity and pigment solubility (12, 29–31). However, it was also found that further temperature increases did not have a significant effect on pigment extraction (32). Therefore, in this case, the use of high temperatures is limited by the high energy demand. On the other hand, it is important to consider that at high temperatures, the ultrasound cavitation effect is weakened. This is because as the temperature rises, the solvent intermolecular forces decrease and their vapor pressure increase causing more solvent vapor enters the cavitation bubbles, which collapse less violently since the vapor contained inside exerts a “cushioning effect” during cavitation (12, 30, 33). This effect will be greater as the temperature approaches the boiling temperature of the solvent. Therefore, if it is required to enhance the non-thermal effects during UAE, the use of low or mild controlled temperatures is recommended, in addition to avoiding deterioration of thermolabile pigments.

#### Pressure

Considering the range of years evaluated in this study, the use of pressures above or below atmospheric pressure has not been studied during the application of the UAE for the extraction of pigments from by-products. However, it was demonstrated that the level of external pressure influences the intensity of the implosion of the cavitation bubbles. For instance, when vacuum pressure was used during ultrasonic processing, cavitation decreased by almost 27% (34), which can reduce ultrasound effects. On the contrary, Raso et al. (33) demonstrated that ultrasound power is increased when pressure is increased above atmospheric.

#### Ultrasound System

For UAE of pigments from by-products, two types of ultrasound devices were used: an ultrasonic bath or an ultrasonic probe. The ultrasonic bath consists of a bath with one or many ultrasonic transducers attached at the bottom. The ultrasonic probe is the most powerful source of ultrasound, working at lower frequencies than ultrasound baths (10, 11) and consists of an ultrasonic transducer attached to a sonic horn (probe) composed of a titanium alloy. This material is thermo-resistant and behaves well under corrosive conditions; however, the erosion of this material is often important since it could represent a health risk (12, 35). In both systems, the by-product sample with their respective solvent can be placed in direct contact with the ultrasound device and in the case of an ultrasonic bath, the by-product and solvent can be placed in a container immersed in a medium to transmit ultrasound waves (usually water).

##### Power

The actual applied acoustic power, which can be determined through hydrophones, aluminum foil method, calorimetric method or chemical method (10, 36, 37) must be considered instead of the nominal power of the equipment or the percentages thereof. Generally, by increasing the ultrasound power until a certain point, higher UAE efficiency could be obtained Montero-Calderon et al. (38). However, at any further increase in acoustic power, compound degradation could occur. This is explained because the cavitation generated during the ultrasound could produce hydroxyl (OH) and hydrogen peroxide (H_2_O_2_) radicals, causing the degradation of compounds (39). In fact, the increase in pigment extraction followed by a decrease as the ultrasonic power continues to increase was reported by Gao et al. (40), Zhang et al. (41), and da Rocha and Noreña (42). Therefore, ultrasound power is a parameter that should be optimized looking for the minimum necessary to obtain the best pigment extraction results and reduce the energy consumption of the UAE process.

##### Frequency and Amplitude

The ultrasound frequency and amplitude influence the cavitation bubble such as their collapse duration and their resonance size. According to Leong et al. (17) ultrasound in the 20–100 kHz frequency region is known to be most effective for extraction. Therefore, both impact the UAE process, where increasing the amplitude can increase the ultrasonic intensity (UI), which refers to the acoustic energy delivered to the sample per unit area of transducer (W/cm^2^) or acoustic energy density (AED), which refers to the acoustic energy delivered per volume of sample (W/mL) (11, 12). In this regard, it was reported the increase in pigment concentration with the increase of ultrasound intensity until a certain level, where at any further increase, the concentration of pigments begins to decrease (43, 44). Therefore, the effect of increasing the amplitude or the ultrasound intensity is similar to the behavior observed for an increase in acoustic power.

#### Solvent

The solvent type and its characteristics influence the UAE, where the choice of each of them depends on their physicochemical properties (viscosity, polarity, pH, and stability), the affinity with the compound to be extracted, the level of toxicity, among other aspects such as renewability and separation capacity. Regarding physicochemical properties, at the low viscosity of solvents, the extraction efficiency of pigments was increased (45). The contrary effect was evidenced at high solvent viscosity (40). Furthermore, Kumar et al. (18) reported that usually for UAE of bioactive compounds from food by-products, solvents such as acidified water, ethanol, other alcohols and their solutions are used. These solvents were usually applied at different concentrations to extract hydrophilic pigments, such as anthocyanins and betalains. For hydrophilic pigments, mixing organic solvents with water increases the extraction yield when compared to pure organic solvents such as alcohols. However, the concentration of water cannot be too high or too low since low yield or purification problems could occur respectively (44, 46, 47).

In addition, as an alternative to the use of volatile organic compounds (acetone, hexane, methanol, and ethanol), other individual or mixture of new compounds were used as solvents in UAE. For example, ionic liquids (40, 48) and different types of vegetable oils such as extra virgin sunflower oil, extra virgin olive oil and refined sunflower oil (49), for lipophilic pigments extraction. On the other hand, for hydrophilic pigments extraction, solvents known as deep eutectic solvents (DES) and natural deep eutectic solvents (NADES) were applied. In fact, these solvents have recently received great attention for their physicochemical features for bioactive compounds extraction from food by-products as an alternative for organic solvents which shows several drawbacks such as toxicity, high volatility and non-renewability (1, 50), were the best selection was based on its price, physicochemical properties, pigment recovery and stability.

#### Sample Matrix

The sample matrix highly influences UAE efficiency, it determines the required optimal processing conditions since for each matrix the necessary UAE conditions are different. It is also important to consider sample preparation operations, according to Vinatoru (51) the pre-treatment of the matrix is important and can impact extraction efficiency. In the case of by-products, in this review, it was noted that since they contain high moisture, they usually are subjected to freezing and/or drying operations, for their conservation. The most used by-products drying methods were convective drying, lyophilization or freeze-drying and drying at room temperature. Then, dried by-products are subjected to grinding operations to reduce their size and increase the contact area with solvent. In fact, it was reported that the pigment extraction may vary depending on the drying method used (32, 52).

While, in the by-products that are not subjected to the drying process, the UAE was carried out using samples in a wet state or after being freeze-thawed without additional size reduction treatment (as in the case of pomace waste) or cut into small pieces (as in the case of peels). Therefore, the type of by-product, its modified structure through freeze-thaw and drying processes and its composition (mainly the pigment to be extracted and compounds that could react with the solvent) should be considered as a starting point in the choice of the other processing parameters levels.

#### Solid-to-Liquid Ratio

The Solid-to-Liquid (S/L) ratio also called SLR (or their reciprocal L/S or LSR) is an important parameter that influences UAE efficiency (18). On the one hand, at a very high S/L ratio, the apparent viscosity of the fluid medium increase hindering both the effect of cavitation and the dispersion of the solvent in the sample. Then, with an initial decrease in S/L ratio, the transport of the solute in solvent increases, as well the apparent viscosity and concentration decrease leading to a greater cavitation effect. Then, at an adequate S/L ratio, the interaction between solid and solvent increases and the ultrasound effects are improved increasing the extraction yield. However, at a very low S/L ratio it could occur a decrease in the yield due to compound degradation, as a consequence of the negative effect of enhanced cavitation. In addition, the disadvantage of using a very low S/L ratio is the high solvent consumption (53). Therefore S/L ratio is another operative parameter to be optimized. Different results regarding the influence of the S/L ratio during the UAE of pigments from by-products were reported, in some cases, authors recommended the use of low values of S/L ratio (54), high values (29, 55), or on the contrary, they reported no effect (43) on the yield of extracted pigments.

#### Time of Processing

**FIGURE 4:**
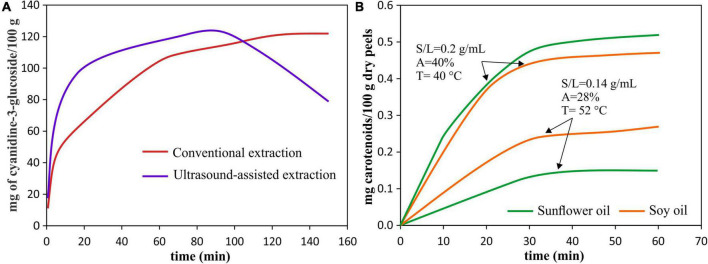
Pigment extraction kinetics. **(A)** Extraction kinetics of anthocyanins from red araçá peel by using UAE [40°C, 40 kHz, 154 W, S/L = 0.2 g/mL] and conventional extraction (90% (v/v) ethanol with 0.1% HCl), data from Meregalli et al. (56). **(B)** Extraction kinetics of carotenoids from pomegranate peels for different UAE conditions: solvents, amplitude (A), temperature (T), and solid-liquid (S/L) ratio, data from Goula et al. (55).

The time of UAE processing is a crucial parameter since, together with power and temperature, it determines energy consumption and extraction efficiency. The main advantages of the UAE application are the acceleration of the extraction process and the increase in extraction yield when compared to a conventional extraction process (Figure 4A) (56).

The time effect is similar to an increase in acoustic power, amplitude and temperature, after reaching a maximum point, further increases in each of these parameters will cause the yield reduction (18). In this sense, a very short extraction time can be insufficient to complete the extraction leaving target compounds in the sample. Initially, the increase in sonication time increases the yield reaching a maximum, at this time, the cavitation effect enhanced the extraction mechanisms. That is, when reaching a maximum yield at a certain time, the equilibrium concentration of the extracted pigment is reached (29, 57). However, further increment in time, the UAE could result in oxidative degradation of the extracted compounds decreasing the yield (41, 56, 58, 59).

The best way to evaluate the effect of processing time on the pigment extraction yield, as well as the influence of other factors (such as type of pigment, type of used solvent, type of sample, power, temperature, amplitude, among others), is by evaluating the kinetics of pigment extraction during UAE (Figure 4). The characteristics of the sample also influence the time required, for example, a larger geometry of the sample will require a longer processing time (60), so the ultrasound causes modifications in the structure that allow the release of intracellular pigments.

Therefore, it is important to highlight that the influence of the mentioned factors does not occur individually, so during the UAE process, it is necessary to control some parameters (as best as possible) as well as the variation of others to obtain optimal conditions in terms of quantity and quality of extracted pigments. The following sections detail the UAE method applied to obtain natural pigments from food by-products.

### Energy Consumption

The UAE process is included in the green extraction methods group. The UAE compared to conventional extraction methods allows the use of a lower amount of solvent, is compatible with the use of green solvents (such as NADES and ILs), increases the extraction yield, improves the quality of the extracted compounds, and reduces the extraction time. These advantages are traduced in lower energy consumption (11, 12, 43, 45, 61–64). However, the energy consumption during a UAE process depends on several factors such as US system (probe or bath device, power, frequency and amplitude level); process application mode (continuous sonoreactor in continuous flow mode or batch reactor); the position of the ultrasound transducers; operation mode (continuous and pulsed operation) and temperature (11, 65). In addition, the energy consumption is influenced not only by the factors mentioned during the UAE processing but also by the use of other techniques associated with the UAE process, for example, the use of enzymes that allows lower energy consumption (66), also by the factors that demand energy after the extraction processing such as during the separation and purification processes. In this regard, for example, the UAE using edible oils as the solvent does not require any subsequent process saving extra power consumption as the pigment-enriched oil can be used directly (67).

Another important aspect that highly influences energy consumption during the UAE process is the plant matrix structure, plasticity and compositional differences (60, 68). For instance, the presence of sensitive compounds requires lower temperature but longer times, while a porous structure requires less time than a compact structure. Consequently, each pigment and matrix, depending on process conditions, shows different extraction behavior demanding a certain amount of energy.

Therefore, energy consumption is specific for each case being necessary for the optimization of UAE processing conditions looking for saving energy.

## Extraction of Natural Pigments With Ultrasound-Assisted Extraction From By-Products

**FIGURE 5:**
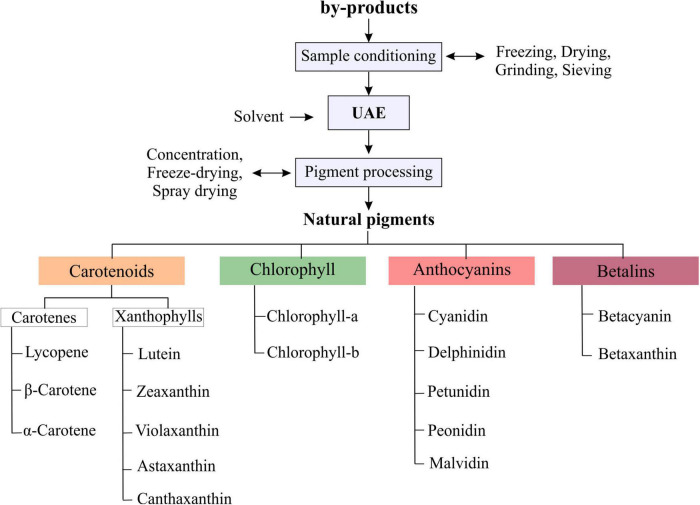
Types of pigments that were obtained from by-products by applying the UAE process.

By-products can be used as a fresh sample or previously conditioned, the sample preparations commonly include freezing or drying by applying hot-air, solar and freeze-drying methods. Then the dried samples are cut or milled and sieved to proceed with UAE. Once the samples are prepared, they are mixed with solvent and subjected to the UAE process, obtaining extracts rich in natural pigments, which can be used in different food, pharmaceutical or cosmetic applications in the form of concentrated extract or purified and dried pigment. The main pigments reported in the literature extracted by UAE from by-products correspond to the large groups of carotenoids, chlorophyll, anthocyanins and betalains (Figure 5).

### Carotenoids

Carotenoids are lipophilic pigments mainly produced by plants, algae, and some animals such as arthropods and salmonids, they are responsible for the characteristic yellowish, orange, and reddish colors. Several types of carotenoids have been identified and categorized into two main groups: xanthophylls (with oxygen in its chemical formula) and carotenes (without oxygen in their chemical formula), carotenogenesis of carotenes and xanthophylls is controlled by the transcript genes, which are mainly regulated by light and temperature (69, 70). Lycopene (C_40_H_56_) is considered the first colored carotenoid in the biosynthesis of many other natural carotenes and xanthophylls (71). From lycopene, α and β carotenes are synthesized, then while α-carotene is converted into lutein, β-carotene is converted into cryptoxanthin, zeaxanthin, antheraxanthin, capsanthin, violaxanthin, and neoxanthin among other (69–71).

Carotenoids are important functional metabolites in the human body because of their antioxidant capacity and for being precursors of vitamin A (72), related to a significant reduction of cancer, cardiovascular diseases, and age-related macular degeneration (73).

### Chlorophyll

Chlorophylls are amphiphilic pigments from photosynthetic organisms, they are distributed in several plants, algae, and cyanobacteria. Two structures of chlorophylls coexist in plants: Chlorophyll-a, which has a methyl (-CH) group at the 7-carbon position and chlorophyll-b, which has an aldehyde (-CHO) group at the 7-carbon position. Due to this structural difference, chlorophylls have a different greenish color, since chlorophyll-a has a blue-green color, while chlorophyll-b shows a blue-yellow color (4, 74, 75). Because the pigment chlorophyll is a naturally abundant compound in various fruits and vegetables and has been consumed since ancient times, its use as a food component is safe. However, the main drawback is its instability, which limits its application as an additive (71).

### Anthocyanins

Anthocyanins are water-soluble pigments that belong to the wider group of polyphenols (57) and are one of the most important flavonoid compounds in plants (76). Over 500 different anthocyanins were identified from several plant sources in their flowers, leaves, and in the seed, pulp or peel of the fruits. The major plant-based anthocyanins are cyanidin, pelargonidin, delphinidin, peonidin, petunidin and malvidin. Anthocyanins (E163) colorants are capable to impart four colors: red, purple, violet, and blue, where pH is a major factor that differentiates the appearance of colors of anthocyanins (4, 5, 77).

Anthocyanins are used as natural pH-dependent colorants in the food industry, they appear to be red or pink in acidic solutions and blue to purple in alkaline conditions. Thus, these can be used for many industrial applications (67). The beneficial health effects of dietary anthocyanins have been widely demonstrated due to their antioxidant activity (67), which means that they have positive effects on the prevention of cardiovascular disease, while they also show antiproliferative, anti-inflammatory, neuroprotective activity and are considered as promising chemoprotective agents in cancer treatment (57).

### Betalains

Betalains are water-soluble nitrogen-containing pigments present in most plants of the order Caryophyllales, which are synthesized from the amino acid tyrosine into two structural groups: the red-violet betacyanins and the yellow-orange betaxanthins. These pigments are composed of a nitrogenous core of betalamic acid, which can either condense with amines to form betaxanthins or with imino compounds to form betacyanins (69, 78, 79). The strength of these colorants is three times higher than anthocyanins (5). Approved as a natural colorant, betalains have been used in different food products like candy, yoghurt, ice cream, salad dressing, powdered drink mix, soft drinks and dessert gelatin (68).

In addition, betalains have been associated with several biological activities such as anti-inflammatory, antiproliferative, antimicrobial activities, and free radical scavenging potential, among others. This was demonstrated through *in vivo* studies, which indicate that betalain supplementations could play a beneficial role in diseases such as hypertension, cancer and dyslipidemia (69, 79, 80).

By considering the results reported by different investigations carried out in the last 5 years, below are described the types of by-products used, the identified pigments, the effect of different factors and processing parameters, among other important results during UAE of natural pigments.

### Extraction of Carotenoids and Chlorophyll

UAE has been used to extract carotenoids from different fruits and vegetables by-products such as acerola, guava, genipap and umbu seeds and peels (72), carrot pomace (73, 81), cantaloupe peel (82), mandarin peel (54, 83, 84), Gac fruit peel (63), mango peel and paste (85), peel of different varieties of orange (38, 43, 48), orange pomace (86), pepper leaves (87), pomegranate peel (55), pumpkin peel (88, 89), purple passion fruit peel (90), sea buckthorn pomace (49, 91, 92), tomato peel and seeds (31, 93–95), and arthropods by-product such as shrimp shell and cephalothorax (40, 96–98). According to these studies, carotenoids can be used as a natural pigment directly in the preparation of various food products such as butter, salad dressing, beverages, ice cream, desserts, candy, bakery, edible oils, meet products, cosmetics, pharmaceutical products, also as an ingredient in feed for aquatic animals and pets.

Supplementary Table 1 shows the experimental and optimum UAE processing conditions of the studies listed above, as well as the types of carotenoids identified in each by-product. The main carotenoids pigments extracted and identified in these by-products were β-carotene, canthaxanthin (trans), lycopene, lutein, zeaxanthin, violaxanthin, and astaxanthin, being β-carotene the most frequent, which was identified in ∼ 45% of by-products. In addition, some by-products are sources of specific carotenoids, for example, in tomato residues, the main carotenoid is lycopene, while in shrimp residues, it is astaxanthin. Among UAE processing conditions, the power level, amplitude level, temperature, extraction time, type and concentration of solvent and solid/liquid ratio were analyzed. The evaluation of different experimental conditions allows identifying the best levels to obtain the maximum yield of carotenoids extraction.

Regarding the types of solvents used to extract carotenoids by UAE, solvents can be classified into conventional organic solvents (COS) and “green solvents”. On the one hand, the COS used were ethyl acetate (63), ethanol, methanol, acetonitrile (73), tetrahydrofuran, butylated hydroxytoluene (85), petroleum ether (95), among others. During carotenoid extraction, considering the polarity of the solvents used is very important. For instance, according to Benmeziane et al. (82), during the UAE of cantaloupe peel, the combination of polar (acetone) with non-polar (hexane) solvents for lipid-soluble compounds extraction seems to enhance the solubilization of the non-polar carotenoids (β-carotene), whereas individual polar solvents (ethanol and acetone) are thought to enhance the solubilization of the polar ones like lutein. Umair et al. (73) mentioned that variation in yield extraction could be because the dissimilar solvent polarities at different concentrations that lead to the increased cell wall permeability, while the use of non-polar solvent (hexane) can help to prevent degradation of heat-sensitive components. Szabo et al. (95) confirmed that the mixture of hexane/acetone lead to obtaining the maximum extraction of carotenoids recovered from tomato by-products. In addition, saponification processes were used to separate the carotenoids from ether bounds, methanolic solution of KOH (20%) was used in these cases (84), also enzymatic pre-treatment with cellulase and pectinase or polygalacturonase has been shown useful to accelerate extraction processes (81, 86).

On the other hand, the explored “green solvents” were d-limonene (43), sunflower, soy, olive and corn oils (55, 88, 92), and ionic liquids (ILs), which have remarkable characteristics and received more attention in the last years. Regarding edible oils, they have been used as extraction solvents to produce enriched oils with carotenoids from sea buckthorn pomace (49). However, it is important to highlight that oils contribute with an initial percentage of carotenoids that may affect the interpretation of the extraction yield obtained, carotenoids in oils are naturally present in greater quantity in the extra virgin olive oil, compared to extra virgin sunflower oil and refined sunflower oil (49), or refined corn oil (92). Particularly, ionic liquids (ILs) are salts with melting temperatures below 100°C, usually composed of a large organic cation and an organic or inorganic anion, that can be described as “designer solvents,” they present chemical and thermal stability, non-flammability, high ionic conductivity, a wide electrochemical potential, high density and low vapor pressure, which facilitate isolation of organic compounds. The used ILs during UAE of carotenoids were, for example, 1-n-butyl-3-methylimidazoliumtetrafluoroborate ([BMIM][BF4]), 1-hexyl-3-methylimidazoliumchloride ([HMIM][Cl]), 1-butyl-3-methylimidazolium chloride ([BMIM][Cl]) and 1-butyl-3-methylimidazolium hexafluorophosphate ([BMIM][PF6]) diluted in alcohol were used to extract carotenoids from orange peel (48). In addition, ILs-in-water microemulsions were used to extract carotenoids from shrimp waste (40).

On the other hand, the factors of temperature, power and processing time are also important during the extraction of carotenoids. In this regard, an increase in temperature from 20 to 40°C resulted in time reduction and an increase in lycopene content obtained by UAE of tomato peels (31). The increasing extraction of total carotenoids at stronger power or ultrasonic intensity was observed by Montero-Calderon et al. (38) where the high ultrasonic power allowed to obtain the maximum of carotenoid extracted from orange peel, and by Boukroufa et al. (43) who reported an increase in carotenoid concentration from citrus fruit waste with the increase of ultrasound intensity until 195 W/cm^2^; however, when intensity is higher than this value, the carotenoid content decreased about 40%. In the same way, Rahimi and Mikani (94) reported that the highest lycopene content (91.49 μg/100 g) was reached at 10 min and high ultrasonic power, while it was reduced until 85.90 μg/100 g, at 18.41 min. In addition, Gao et al. (40) reported that astaxanthin extracted from shrimp waste increased with used power from 30 W to 50 W, but when power was increased to 90 W, declined. Finally, regarding the UAE time, as observed in Figure 4B, the extraction yield of carotenoids from pomegranate peels along the UAE depends not only on time but also on the used solvent, the amplitude, temperature, and solid-liquid ratio (55). The same was observed by Chutia and Mahanta (90), where to extract carotenoids from passion fruit peel, a different optimum time was needed depending on if it was used olive oil or sunflower oil as solvents. Therefore, as explained in sections “Temperature,” “Ultrasound System,” and “Time of Processing,” the effects are not proportional, and high temperature, ultrasonic intensity and long times can lead to the pigment degradation.

For the S/L ratio, it has been shown that the carotenoid extraction yield increases, decreases or does not change. For example, Ordóñez-Santos et al. (54) evaluated the S/L ratios of 0.4–1.2 g/L to extract carotenoids from mandarin epicarp, where after optimization, the ratio of 0.4 g/L allowed to obtain a maximum extraction of β-carotene. In addition, carotenoid extraction yield increased at S/L of 20 g/L but decreased at a higher S/L ratio. In contrast, the increase of the S/L ratio from 100 to 200 g/L does not have a particular impact on the extraction yield of carotenoids in citrus fruits waste (43). The main reason for this effect is that a lower S/L ratio could cause greater concentration differences between phases which accelerated the carotenoid diffusion into the solvent, but also the ultrasound effects could be improved at lower S/L ratios (84, 90).

In addition to providing color, the carotenoid-rich extracts obtained through UAE exhibited antioxidant activity (49, 82) and peroxyl radical scavenging activity (48). Therefore, carotenoids can be highlighted as a promising natural colorant for application mainly in the food industry (97), also as an ingredient owning healthy components for the development of functional foods (92, 98). Extracted carotenoids can be used in the form of enriched edible oils (91), or can be integrated into high water content food products as a spray-dried powder (95). However, the carotenoids use can be limited by some interactions with other compounds in food products. For instance, the flavor and composition of some edible oils can be affected; while metals, acid value and the percentage of conjugated dienes in edible oils can be responsible for the oxyradical species (55). Also, the thermal stability of extracted carotenoids can limit the industrial use, Murador et al. (48) noted losses of 91.1% (at 60°C) and 100% (at 90°C) of all-trans-violaxanthin and losses of 89.1–98.3% for 9-cis-violaxanthin presented in ILs extract, all-trans-lutein was the most stable with losses of 35.2–48.8% at the same temperatures respectively. These results confirm violaxanthin to be the most unstable carotenoid to heating, while lutein was more stable.

Regarding the extraction of chlorophyll from by-products with UAE, there are very few studies carried out, in the last 5 years only 4 works were found, of which one is a conference abstract (99) and other used UAE as part of the analytical method (100). All studies evaluated chlorophyll extraction from leaves (agricultural waste produced during harvest). Chlorophyll was extracted by UAE from the powder of the dry leaves of carrot (99), papaya (101), cassava (100), and olive (102). Among the processing conditions performed in the mentioned studies, ultrasound probe and bath were used, a maximum temperature of 50°C, extraction times from 5 min to 90 min, S/L ratio from 0.033 to 0.05 g/mL using as solvents water, hexane, acetone, or a mixture of water with ethanol. According to Molina et al. (99) the UAE proved to be more efficient than maceration extraction and compared to water and hexane, ethanol allowed the extraction of greater amounts of chlorophylls. For instance, Zulqarnain et al. (101) determined that the optimal conditions for chlorophyll extraction from papaya leaf powder were ethanol 80%, 5 min, 35°C and 1 g of raw material, obtaining a chlorophyll yield of 40% (0.4 g/g) in the extract, which in the purified form was composed of chlorophyll-a (14.125 mg/g) and chlorophyll-b (19.845 mg/g). In addition, it was obtained chlorophyll-a (1.3 mg/g of freeze-dried extract) and chlorophyll-b (0.54 mg/g of freeze-dried extract) from olive leaves through UAE application (102), while in cassava leaves, it was obtained that the total chlorophyll (sum of chlorophyll-a and chlorophyll-b) content of the cassava leaves ranged from 326.27 to 747.86 mg/100 g dry matter (100).

In summary, as an alternative to conventional extraction, in recent years researchers have been working on the development of alternative UAE processes using “greener solvents” such as edible oils or ILs with more sustainable credentials. In addition, it has been observed that the carotenoid and chlorophyll extraction conditions using UAE must be studied and optimized for each type of by-product. Therefore, other types of by-products and conditions should be better explored, especially for chlorophyll extraction since there are very few studies to date.

### Extraction of Anthocyanins

UAE have been used to extract anthocyanins from different fruits and vegetables by-products such as bilberry press cake (76), blackberry bagasse and pulp precipitate (39, 67), blueberry peel (57, 67), grape pomace (32, 42, 45, 103, 104), eggplant peel (60, 62, 105), fig peel (106), jabuticaba eel (44, 107, 108), mulberry wine residues (41), peach waste (Plazzotta) (52), pomegranate peel (109, 110), purple corn bran (111), raspberry wine residues (112), red araçá (56), sweet cherries peel (113), wine lees (61), and huajiao peel (114). According to the reviewed authors, extracted anthocyanins can be used in the food industry such as bakery products, functional foods, and pharmaceutical and cosmetic fields, as an alternative to synthetic food colorants, also as a microencapsulated powder in yoghurt, and marshmallows.

Supplementary Table 2, for the works listed above, shows the UAE processing conditions and the optimum levels, as well as the types of anthocyanins identified in each by-product. The main anthocyanins extracted and identified were Galactoside derivates such as Delphinidin-3-galactoside, Cyanidin-3-galactoside, Petunidin-3-galactoside, Peonidin-3-galactoside, Malvidin-3-galactoside; Glucoside derivates such as Delphinidin-3-glucoside, Cyanidin-3-glucoside, Petunidin-3-glucoside, Peonidin-3-glucoside, Malvidin-3-glucoside, Malvidin-3-glucoside; Arabinoside derivates such as Delphinidin-3-arabinoside, Cyanidin-3-arabinoside, Petunidin-3-arabinoside, Peonidin-3-arabinoside, Malvidin-3-arabinoside; Rutinoside derivates such as Cyanidin-3-rutinoside, Delphinidin-3-rutinoside; Petunidin-3-rutinoside; Acetylgalactoside derivates such as Delphinidin-3-(6-acetyl)-galactoside, Cyanidin-3-(6-acetyl)-galactoside, Petunidin-3-(6-acetyl)-galactoside, Peonidin-3-(6-acetyl)-galactoside, Malvidin-3-(6-acetyl)-galactoside; Acetylglucoside derivates such as Cyanidin-3-(6-acetyl)-glucoside; Acetylhexoside derivates such as Cyanidin-3-acetyl-hexoside; Rutinoside-glucoside derivates such as Delphinidin-3-rutinoside-5-glucoside, Malvidin-3-rutinoside-5-glucoside; and Coumaroyl derivates Peonidin-3-(6-O-p-coumaroyl) and Malvidin-3-(6-O-p-coumaroyl).

The conditioning stage of the by-products has an important effect on the extraction of anthocyanins. For example, the anthocyanin extraction from grape pomace by UAE was enhanced more significatively in freeze-dried by-product samples compared to fresh ones (32). In contrast, anthocyanins were not detected in the dried peach waste sample compared to the frozen sample (52). On the other hand, among the evaluated UAE experimental conditions are the temperature, type and concentration of solvent and S/L ratio, power level and extraction time.

Regarding the temperature, a higher extraction yield was observed in anthocyanin obtained from wine lees when the temperature of extraction increased from 25 to 35°C (29). However, González et al. (32) found that increasing the temperature from 15 to 60°C did not have a significant effect on the UAE of anthocyanins from grape pomace. This reinforces the fact that the use of high temperatures, mainly above 50°C, can be detrimental. Different solvents were used to extract anthocyanins with UAE, where distilled, purified or deionized water has been commonly used in 44% of cases, either at neutral pH (26% of cases), acidic pH = 1.5–3.5 (26% of cases, by using citric acid or chloride acid) or basic pH = 12 with NaOH (4% of cases) being optimum the use of acidified water. Other commonly used solvents are hydroalcoholic solutions like ethanol (67% of cases) almost acidified, methanol (11% of cases), isopropanol (7.4% of cases), and hydrochloric acid solution (7.4% of cases). Specifically, it was recommended the use of aqueous ethanol solution (50% v/v) to extract anthocyanins from wine lees (29) and jabuticaba by-products (44), or acidified ethanol to extract anthocyanin from eggplant peels (60). In addition, Machado et al. (67), compares the yield extraction using acidified water (pH 2.0) and aqueous ethanol solutions (50, 70% v/v) obtaining that 70% v/v hydroalcoholic solution allows the best yield extraction. Dranca and Oroian (105) compare the use of ethanol, methanol and propanol, obtaining the highest recovery with methanol solution. On the other hand, NADES were also applied (11% of cases), as emerging green solvents. For instance, for the UAE of anthocyanins from blueberry peels (57), and grape pomace (45), it was applied different NADES. Paniæ et al. (45) compared the use of NADES with acidified ethanol solution (70% v/v) obtaining similar effects for ChCit (Choline Chloride:Citric acid) and ChProMa (Choline:Proline:Malic acid) at pH 0.49–3.27. Anthocyanins are highly polar compounds that are better solubilized in polar than in non-polar solvents, and their stability depends on pH value, they are stable at pH 2.0 and are degraded at pH > 7.0 (61). Acid-based NADES have polarities similar to water and at concentrations of 25% v/v decrease their viscosity improving the mass transfer during the UAE of anthocyanins from grape pomace (45).

Regarding the S/L ratio, Goula et al. (55) evaluated the S/L ratios from 100 to 0.333 g/L, being the ratio of 100 g/L the necessary for optimum conditions, while the anthocyanin extraction from wine lees slightly decreased as the S/L ratio decreased from 100 to 25 g/L (29). More and Arya (110) and Chen et al. (111) studied this factor at 20–100 g/L and 28.6–66.7 g/L respectively. All these studies demonstrated that the increase of the S/L ratio decrease the yield extraction, this agrees with the expected mass transfer phenomenon, where at a higher gradient between solid and solvent, the driving force during the mass transfer is greater. Nevertheless, a lower S/L ratio may cause more solvent consumption, so also the optimum ratio needs to be investigated for each case.

Concerning ultrasound power intensity and processing time effects during the extraction of anthocyanins, it has been shown that when ultrasonic power increased up to 300 W, it was reached the maximum of anthocyanin content extracted from mulberry wine residues; however, a further increase in ultrasonic power decreased the yield (41). The same behavior was observed for anthocyanins extracted from jabuticaba peel increased as the ultrasound intensity increased from 1.1 to 7.3 W/cm^2^, but at 13 W/cm^2^ the anthocyanins decreased (44). In contrast, in some cases the power level variation also may not affect pigment extraction, this was observed by da Rocha and Noreña (42) during UAE of anthocyanins from grape pomace. Regarding time, in samples with higher geometry, a longer extraction time is usually required to cause cell disruption and increase the release of pigments, this was observed during anthocyanin extraction from eggplant peel cut into squares (60). However, in powdered samples, too large extraction times can cause a reduction in yield (39). In fact, as observed in Figure 4A, this behavior was reported in anthocyanins extracted from red araçá peel (56) and mulberry wine residues (41) were after reaching the highest anthocyanin content at 90 min, after this time their content decreased, suggesting degradation. Furthermore, any variation in anthocyanin concentration after a certain processing time also was reported. This was observed during the extraction of anthocyanin from wine lees, where after 15 min a steady anthocyanin concentration was achieved (29). In the same manner, during UAE of anthocyanins from blueberry peels, at 30 min the equilibrium concentration was reached (57). In addition, Varo et al. (76) noted that the highest extraction yield was reached at approximately between 5 and 7 min using UAE, and became constant at 15–20 min, so increasing times beyond these values would not be necessary as it could degrade the pigment. The possible reasons given for these behaviors have been described in sections “Ultrasound System” and “Time of Processing”; however, usually, the maximum power used can achieve the highest yield in a short time.

Regarding characteristics reported in anthocyanin extracts, the instrumental color was the most reported. The range of color parameters L* (lightness), a* (redness: green to red) and b* (yellowness: blue to yellow) obtained were L*: 5.09–79.7; a*: 21.97–70.9 and b*: 7.39–36.01 (42, 44, 76). Some variations in the behavior of color parameters have been reported in some cases during processing. Among them, by increasing processing time, L* decreases, while a* and b* increase. Otherwise, L* and b* decrease, and a* increases when the water/ethanol ratio increases. The decrease in L* parameter means that the extracts become darker, with the a* increase and b* decrease, red color was established. This inverse behavior between a* and b* parameters is correlated with the high content of anthocyanins. In contrast, lower values of L* but higher values of a* and b* are related to brownish color as a possible consequence of the pigment degradation. On the other hand, the decrease of all parameters indicates the loss of red color, this behavior was related to high ultrasound intensities and the decrease of bioactive compounds and their antioxidant capacity (44).

In summary, evaluation of anthocyanin extraction kinetics by UAE is important as well as optimization studies since the different types of solvents and their characteristics, as well as the different levels of power, S/L ratio, temperature and time can produce results of increase, decrease or no variation in the yield of extracted anthocyanins. Furthermore, few studies have evaluated and/or proposed options to improve the stability of anthocyanins extracted by the UAE process from by-products. In this regard, the encapsulation of anthocyanins in whey protein isolate and chitosan (113) and alginate-Ca^2+^ (103), were evaluated. Bruno Romanini et al. (103) recommended the encapsulated anthocyanins for future food applications since it protects anthocyanins against light. In fact, anthocyanin complex with biomacromolecules (such as proteins) has the potential to enhance the thermal stability, oxidative stability and photostability of anthocyanin extracts preventing anthocyanin degradation during food processing (115–117). Therefore, the stability and interaction with other compounds need to be implemented in studies of pigments extracted by the UAE process.

### Extraction of Betalains

UAE has been applied to obtain extracts rich in betalains from by-products such as beetroot peel and pomace (46, 118), prickly pear peel (47), pitahaya or dragon fruit peel (119), quinoa seed husks (68), and harvest waste such as amaranth flowers (120) and beet leaves (121). Supplementary Table 3 shows the processing conditions by UAE of the works listed above, as well as the types of betalains identified in each by-product. The amount of betalain present in the by-product can be less than, equal to or greater than that in the edible part of the raw material. For example, Fernando et al. (46) showed that the yield of betalains in beet residue is equivalent to the content in the whole beet or beet juice.

Among the experimental conditions evaluated for UAE extraction are the power level, amplitude level, extraction time, type and concentration of solvent and S/L ratio. Adequate control of these factors allows for obtaining the best extraction yield and betalain content. For instance, Melgar et al. (47) during the UAE of opuntia fruit peels obtained the highest total betacyanin content (197.51 mg/g) by applying a time of 1.5 min, S/L ratio of 5 g/L, methanol 50% at 20°C. In contrast, the lowest total betacyanin content (72.01 mg/g) was obtained at the processing conditions of 1.5 min, 25 g/L, methanol 100% and 20°C. This demonstrates that the S/L ratio must be adequate to guarantee the non-degradation of the betalains as well as the non-saturation of the solvent. On the other hand, the concentration of the ideal solvent is related to the affinity of the pigment. According to Fernando et al. (46), since betalains are hydrophilic pigments; the mixture of organic solvents with water increases the extraction yield when compared to pure organic solvents such as alcohols (ethanol, methanol, among others). However, although pure water can improve the betalain yield, it has caused difficulties during the solute separation by filtration due to coextraction of mucilaginous compounds such as pectin Considering this, solvents such as methanol 50% to extract betalains from opuntia peels (47), or ethanol 30% to extract betalains from beetroot waste (46) were recommended. On the other hand, the factors of power and processing time are also important during the extraction of betalains. It has been shown that up to a certain point there is an increase in the concentration of pigments extracted as the power or process time is increased; however, if the power and time are too high, they can lead to the deterioration of the extracted pigment. In this regard, Šeremet et al. (118), after 30 min of UAE processing, reported an amount of betaxanthin of 8.61 ± 0.08 mg vulgaxanthin I/g dry matter (dm), which after 60 min decreased to 6.98 ± 0.06 mg vulgaxanthin I/g dm. Therefore, depending on the extraction matrix, it should be evaluated whether it is suitable to use pure water or a combination of water with organic solvents in percentages not greater than 50%, also the required ultrasound power and processing time should be optimized for each by-product type.

In addition to providing color, the betalain rich extracts obtained through UAE present antioxidant and antibacterial properties. Therefore, it can be highlighted as a promising natural colorant for application mainly in the food sector as an alternative to synthetic food colorants (46, 119, 120). However, compared to synthetic colorants, their wider use could be greatly limited by their poor stability during processing and storage. Laqui-Vilca et al. (68) evaluated the thermal stability (at 90°C) of betalains extracted by UAE from husks of the quinoa seeds, reporting that the betalains from husks of quinoa “Bright red Pasankalla” showed higher stability (first-order rate constants (k) of 0.019 ± 0.008 min^–1^ and half-life (t_1/2_) of 37 min) than those from “Red Pasankalla” quinoa samples, but both presented thermal stability similar to betalains from beetroot. On the other hand, Fernando et al. (46) evaluated the betalain stability, reporting that during the 4-week storage, betalains quickly degraded at room temperature in contrast to -20°C. Betalain degradation is evidenced by a marked reduction of a* values and increased values of b* (i.e. reduction of red color and increase of yellow color) along with the storage. Although, studies that have evaluated the stability of betalains are still scarce and the stability of betalains from other by-products is not known.

Finally, it is important to mention that during the extraction of betalains, some processing conditions have not been clearly defined. For example, not all studies report the type of ultrasound device used as well as the set temperature of processing or the temperature reached during processing. These factors are important since the type of ultrasound equipment and its characteristics, as well as the temperature, reached during the process, strongly influence the quality of extraction using UAE (12), as was described in section 2.2. In addition, more studies are recommended to better explore the betalain purification and bioactivity, as well as their physical stability evaluation inside and outside a food matrix. In addition, new studies are required to explore the optimal processing conditions in other by-products rich in betalains.

## Conclusion and Future Perspectives

In the last 5 years, the UAE of natural pigments from food processing by-products has been increasingly motivated by the increasing generation of residues, environmental concerns, the advantages of ultrasound technology, and the beneficial properties of natural pigments in health. In this review, it was found that the main pigments extracted from by-products were carotenoids and anthocyanins with 43 and 40%, respectively, followed by betalains (11%) and finally chlorophyll (6%). The most used by-products to extract pigments were peels from different fruits and vegetables with 33%, followed by pomace (a ground waste made up of skins, seeds and pulp precipitates) with 16%, while the minority percentages were leaves and flowers (as harvest waste), shrimp shell and cephalothorax, grain husk, among others. The by-products used came from different sources, mostly of plant origin, mainly citrus (11%), grape (9%), tomato (6%), among others, while in the case of carotenoids, in addition to plant sources, a well-studied source has been shrimp by-products (6%).

Compared to conventional extraction processes, the main advantages reported with the use of UAE for pigment extraction from by-products are:

– Increases the yield and quality of extracted pigment.
– Allows less processing time required to reach the maximum extraction.
– Promotes mass transfer allowing the use of less solvent and the use of “green” solvents such as water, edible oils, ionic liquids and NADES.
– Environmentally friendly technology with low carbon dioxide emissions.

However, disadvantages have also been reported, mainly related to the degradation of pigments under severe conditions, that is, processing at high temperatures, high power and/or amplitude for long processing times. Possible reasons given for the decrease in extracted pigments after a certain point of energy delivered to the system during UAE include: the effect of free radicals generated during acoustic cavitation (39), the generation of a greater number of bubbles at high acoustic intensities that could hinder the propagation of shock waves but also may coalesce, imploding faintly and causing a reduction in the cavitation effect (43). Therefore, it is still unclear and is necessary to better describe the mechanisms by which pigments are degraded after a certain point of UAE processing.

Another important aspect, little explored, is the energy consumption during the UAE process compared to other non-conventional extraction methods. Some comparisons were made regarding microwave-assisted extraction (MAE), where UAE demands less energy to extract thermolabile compounds at room temperature (64) but compared to other non-conventional methods, the UAE could be less efficient. However, the calculation of energy consumption is complex and the level of consumption cannot be generalized since it depends on many factors indicated in section 2.3. Therefore, this aspect should be better explored in future studies where the specific energy consumption (SEC) could be considered to make comparisons between treatments or processes.

Among the process conditions that substantially influence the results by applying UAE, are the concentration and properties of the solvent, the structure, composition of the sample and the previous conditioning (drying method, milling or cutting level, for example), S/L ratio, those related to ultrasound technology (power, frequency, amplitude), temperature and process time. Regarding the processing conditions, those related to factors of solvent, sample and process time have been well controlled and specified in the studies. On the contrary, some weaknesses have been observed regarding the conditions related to ultrasound technology and temperature control. Among them, it has been found that the nominal power of the ultrasound equipment is usually reported, and based on this value, subsequent calculations are made either as a percentage or as an acoustic intensity, without previously applying a method to calculate the real energy delivered to the system. It is worth mentioning that the ultrasonic nominal power is substantially higher than the actual (measured) power absorbed by the medium, including a non-linear behavior (122) that is, it is not proportional. On the other hand, in most cases, the process temperature is not controlled, so it is not possible to differentiate the non-thermal effects of ultrasound since they can be jeopardized by the thermal effect. Consequently, future studies are encouraged to calculate the actual ultrasound power and to control the temperature during the extraction process, in order to better understand the mechanisms as well as to be able to effectively compare the results.

Finally, although chlorophyll has great potential for use as a natural food colorant the information available on chlorophyll extraction by using UAE is scarce. Therefore, different conditions of applying UAE to agricultural by-products such as leaves or bark that are generated in large quantities during the harvest of fruits and vegetables remain to be explored. In addition, future studies are recommended to better explore anthocyanin, carotenoid, chlorophyll and betalain purification, concentration and/or encapsulation, as well as studies to better explore the purification, and their bioaccessibility, bioavailability and bioactivity. In addition, interesting studies are recently being carried out to evaluate the stability and interaction of anthocyanins with biomolecules, which may have a protective effect against pigment deteriorating conditions (117). In this sense, in pigments extracted by UAE from by-products, future studies are recommended to evaluate the stability during processing and the interaction with food matrix compounds. On the other hand, new studies are required to explore and optimize processing parameters but also the evaluation of other by-products sources rich in natural pigments.

## Author Contributions

GL collected and organized the information from the databases, wrote sections of the first draft of the manuscript. MR contributed to the conception and design of the study, collected and organized the information from the databases, wrote sections of the first draft of the manuscript. Both authors contributed to manuscript revision, read, and approved the submitted version.

## Conflict of Interest

The authors declare that the research was conducted in the absence of any commercial or financial relationships that could be construed as a potential conflict of interest.

## Publisher’s Note

All claims expressed in this article are solely those of the authors and do not necessarily represent those of their affiliated organizations, or those of the publisher, the editors and the reviewers. Any product that may be evaluated in this article, or claim that may be made by its manufacturer, is not guaranteed or endorsed by the publisher.

## References

[1] Gullón P Gullón B Romaní A Rocchetti G Lorenzo JM. Smart advanced solvents for bioactive compounds recovery from agri-food by-products: a review. *Trends Food Sci Technol.* (2020) 101:182–97. 10.1016/j.tifs.2020.05.007

[2] Wani FA Rashid R Jabeen A Brochier B Yadav S Aijaz T Valorisation of food wastes to produce natural pigments using non-thermal novel extraction methods: a review. *Int J Food Sci Technol.* (2021) 56:4823–33. 10.1111/ijfs.15267

[3] Cano-Lamadrid M Artés-Hernández F. By-products revalorization with non-thermal treatments to enhance phytochemical compounds of fruit and vegetables derived products: a review. *Foods.* (2022) 11:59. 10.3390/foods11010059 35010186 PMC8750753

[4] Sharma M Usmani Z Gupta VK Bhat R. Valorization of fruits and vegetable wastes and by-products to produce natural pigments. *Crit Rev Biotechnol.* (2021) 41:535–63. 10.1080/07388551.2021.1873240 33634717

[5] Ramesh M Muthuraman A. Chapter 1 - Flavoring and Coloring Agents: Health Risks and Potential Problems. In: Grumezescu AM Holban AM editors. *Natural and Artificial Flavoring Agents and Food Dyes.* Cambridge, MA: Academic Press (2018). p. 1–28. 10.1016/b978-0-12-811518-3.00001-6

[6] Carpentieri S Soltanipour F Ferrari G Pataro G Donsì F. Emerging green techniques for the extraction of antioxidants from agri-food by-products as promising ingredients for the food industry. *Antioxidants.* (2021) 10:1417. 10.3390/antiox10091417 34573049 PMC8471374

[7] Young HD Freedman RA. *University Physics with Modern Physics.* 14th ed. London: Pearson (2015).

[8] Gallego-Juárez JA. Basic Principles of Ultrasound. In: Villamiel M Montilla A García-Pérez JV Cárcel JA Benedito J editors. *Ultrasound in Food Processing.* Hoboken, NJ: Wiley Online Books (2017).

[9] Kentish SE. Chapter 1 - Engineering Principles of Ultrasound Technology. In: Bermudez-Aguirre D editor. *Ultrasound: Advances for Food Processing and Preservation.* Amsterdam: Elsevier (2017). p. 1–13. 10.1016/b978-0-12-804581-7.00001-4

[10] Mason TJ Peters D. *Practical Sonochemistry: Power Ultrasound Uses and Applications.* Sawston: Horwood Publishing Limited (2004).

[11] Tiwari BK. Ultrasound: a clean, green extraction technology. *Trends Anal Chem.* (2015) 71:100–9. 10.1016/j.trac.2015.04.013

[12] Chemat F Rombaut N Sicaire A-G Meullemiestre A Fabiano-Tixier A-S Abert-Vian M. Ultrasound assisted extraction of food and natural products. Mechanisms, techniques, combinations, protocols and applications. A review. *Ultrason Sonochem.* (2017) 34:540–60. 10.1016/j.ultsonch.2016.06.035 27773280

[13] Hamdaoui O Naffrechoux E. An investigation of the mechanisms of ultrasonically enhanced desorption. *AIChE J.* (2007) 53:363–73. 10.1002/aic.11090

[14] Yasui K. Chapter 3 - Dynamics of Acoustic Bubbles. In: Grieser F Choi P-K Enomoto N Harada H Okitsu K Yasui K editors. *Sonochemistry and the Acoustic Bubble.* Amsterdam: Elsevier (2015). p. 41–83. 10.1016/b978-0-12-801530-8.00003-7

[15] Gould RK. Rectified diffusion in the presence of, and absence of, acoustic streaming. *J Acoust Soc Am.* (1974) 56:1740–6. 10.1121/1.397192 3209776

[16] Mason TJ Lorimer JP. *Applied sonochemistry: The Uses of Power Ultrasound in Chemistry and Processing.* Weinheim: Wiley-VCH (2002).

[17] Leong T Juliano P Knoerzer K. Advances in ultrasonic and megasonic processing of foods. *Food Eng Rev.* (2017) 9:237–56. 10.1007/s12393-017-9167-5

[18] Kumar K Srivastav S Sharanagat VS. Ultrasound assisted extraction (UAE) of bioactive compounds from fruit and vegetable processing by-products: a review. *Ultrason Sonochem.* (2021) 70:105325. 10.1016/j.ultsonch.2020.105325 32920300 PMC7786612

[19] Wu TY Guo N Teh CY Hay JXW. Theory and fundamentals of ultrasound. In: Wu TY Guo N Teh CY Hay JXW editors. *Advances in Ultrasound Technology for Environmental Remediation*. Heidelberg: Springer (2013). p. 5–12.

[20] Bhaskaracharya R Kentish S Ashokkumar M. Selected applications of ultrasonics in food processing. *Food Eng Rev.* (2009) 1:31–49. 10.1007/s12393-009-9003-7

[21] Petigny L Périno-Issartier S Wajsman J Chemat F. Batch and continuous ultrasound assisted extraction of Boldo leaves (*Peumus boldus* Mol.). *Int J Mol Sci.* (2013) 14:5750–64. 10.3390/ijms14035750 23481637 PMC3634473

[22] Pourcel L Irani NG Lu Y Riedl K Schwartz S Grotewold E. The formation of anthocyanic vacuolar inclusions in *Arabidopsis thaliana* and implications for the sequestration of anthocyanin pigments. *Mol Plant.* (2010) 3:78–90. 10.1093/mp/ssp071 20085894 PMC2807924

[23] Sadali NM Sowden RG Ling Q Jarvis RP. Differentiation of chromoplasts and other plastids in plants. *Plant Cell Rep.* (2019) 38:803–18. 10.1007/s00299-019-02420-2 31079194 PMC6584231

[24] Floros JD Liang H. Acoustically assisted diffusion through membranes and biomaterials. *Food Technol.* (1994) 48:79–84.

[25] Legay M Gondrexon N Le Person S Boldo P Bontemps A. Enhancement of heat transfer by ultrasound: review and recent advances. *Int J Chem Eng*. (2011) 2011:670108. 10.1155/2011/670108

[26] Miano AC da Costa Pereira J Miatelo B Augusto PED. Ultrasound assisted acidification of model foods: kinetics and impact on structure and viscoelastic properties. *Food Res. Int.* (2017) 100:468–76. 10.1016/j.foodres.2017.07.045 28873710

[27] Miano AC Ibarz A Augusto PED. Mechanisms for improving mass transfer in food with ultrasound technology: describing the phenomena in two model cases. *Ultrason Sonochem.* (2016) 29:413–9. 10.1016/j.ultsonch.2015.10.020 26585022

[28] Toma M Vinatoru M Paniwnyk L Mason TJ. Investigation of the effects of ultrasound on vegetal tissues during solvent extraction. *Ultrason Sonochem.* (2001) 8:137–42. 10.1016/s1350-4177(00)00033-x 11326609

[29] Romero-Díez R Matos M Rodrigues L Bronze MR Rodríguez-Rojo S Cocero MJ Microwave and ultrasound pre-treatments to enhance anthocyanins extraction from different wine lees. *Food Chem.* (2019) 272:258–66. 10.1016/j.foodchem.2018.08.016 30309541

[30] Santos HM Lodeiro C Capelo-Martinez J-L. *The Power of Ultrasound in Ultrasound in Chemistry: Analytical Applications.* Weinheim: Wiley-VCH, (2009).

[31] Tsvetko P Milena N Donka T. Improved carotenoid extraction from Bulgarian tomato peels using ultrasonication. *Ann Univ Dunarea Jos Galati.* (2017) 41:41–9.

[32] González M Barrios S Budelli E Pérez N Lema P Heinzen H. Ultrasound assisted extraction of bioactive compounds in fresh and freeze-dried Vitis vinifera cv Tannat grape pomace. *Food Bioprod Process.* (2020) 124:378–86. 10.1016/j.fbp.2020.09.012

[33] Raso J Mañas P Pagán R Sala FJ. Influence of different factors on the output power transferred into medium by ultrasound. *Ultrason Sonochem.* (1999) 5:157–62. 10.1016/s1350-4177(98)00042-x 11269955

[34] Miano AC Rojas ML Augusto PED. Combining ultrasound, vacuum and/or ethanol as pretreatments to the convective drying of celery slices. *Ultrason Sonochem.* (2021) 79:105779. 10.1016/j.ultsonch.2021.105779 34649164 PMC8517379

[35] Mawson R Rout M Ripoll G Swiergon P Singh T Knoerzer K Production of particulates from transducer erosion: implications on food safety. *Ultrason Sonochem.* (2014) 21:2122–30. 10.1016/j.ultsonch.2014.04.005 24815104

[36] Contamine RF Wilhelm AM Berlan J Delmas H. Power measurement in sonochemistry. *Ultrason Sonochem.* (1995) 2:S43–7.

[37] Makino K Mossoba MM Riesz P. Chemical effects of ultrasound on aqueous solutions. Formation of hydroxyl radicals and hydrogen atoms. *J Phys Chem.* (1983) 87:1369–77. 10.1021/j100231a020

[38] Montero-Calderon A Cortes C Zulueta A Frigola A Esteve MJ. Green solvents and ultrasound-assisted extraction of bioactive orange (*Citrus sinensis*) peel compounds. *Sci Rep.* (2019) 9:16120. 10.1038/s41598-019-52717-1 31695137 PMC6834654

[39] Zafra-Rojas QY González-Martínez BE Cruz-Cansino NDS López-Cabanillas M Suárez-Jacobo Á Cervantes-Elizarrarás A Effect of ultrasound on in vitro bioaccessibility of phenolic compounds and antioxidant capacity of blackberry (*Rubus fruticosus*) Residues cv. Tupy. *Plant Foods Hum Nutr.* (2020) 75:608–13. 10.1007/s11130-020-00855-7 33006130

[40] Gao J You J Kang J Nie F Ji H Liu S. Recovery of astaxanthin from shrimp (*Penaeus vannamei*) waste by ultrasonic-assisted extraction using ionic liquid-in-water microemulsions. *Food Chem.* (2020) 325:126850. 10.1016/j.foodchem.2020.126850 32387959

[41] Zhang L Fan G Khan MA Yan Z Beta T. Ultrasonic-assisted enzymatic extraction and identification of anthocyanin components from mulberry wine residues. *Food Chem.* (2020) 323:126714. 10.1016/j.foodchem.2020.126714 32334321

[42] da Rocha CB Noreña CPZ. Microwave-assisted extraction and ultrasound-assisted extraction of bioactive compounds from grape pomace. *Int J Food Eng.* (2020) 16:20190191. 10.3389/fbioe.2020.00645 32671043 PMC7333169

[43] Boukroufa M Boutekedjiret C Chemat F. Development of a green procedure of citrus fruits waste processing to recover carotenoids. *Resour Effic Technol.* (2017) 3:252–62. 10.1016/j.reffit.2017.08.007

[44] Gadioli Tarone A Keven Silva E Dias de Freitas Queiroz Barros H Baú Betim Cazarin C Roberto Marostica Junior M. High-intensity ultrasound-assisted recovery of anthocyanins from jabuticaba by-products using green solvents: effects of ultrasound intensity and solvent composition on the extraction of phenolic compounds. *Food Res Int.* (2021) 140:110048. 10.1016/j.foodres.2020.110048 33648273

[45] Paniæ M Gunjeviæ V Cravotto G Radojèiæ Redovnikoviæ I. Enabling technologies for the extraction of grape-pomace anthocyanins using natural deep eutectic solvents in up-to-half-litre batches extraction of grape-pomace anthocyanins using NADES. *Food Chem.* (2019) 300:125185. 10.1016/j.foodchem.2019.125185 31326673

[46] Fernando GSN Wood K Papaioannou EH Marshall LJ Sergeeva NN Boesch C. Application of an ultrasound-assisted extraction method to recover betalains and polyphenols from red beetroot waste. *ACS Sustain Chem Eng.* (2021) 9:8736–47. 10.1021/acssuschemeng.1c01203

[47] Melgar B Dias MI Barros L Ferreira ICFR Rodriguez-Lopez AD Garcia-Castello EM. Ultrasound and microwave assisted extraction of opuntia fruit peels biocompounds: optimization and comparison using RSM-CCD. *Molecules.* (2019) 24:3618. 10.3390/molecules24193618 31597259 PMC6804160

[48] Murador DC Braga ARC Martins PLG Mercadante AZ de Rosso VV. Ionic liquid associated with ultrasonic-assisted extraction: a new approach to obtain carotenoids from orange peel. *Food Res Int.* (2019) 126:108653. 10.1016/j.foodres.2019.108653 31732025

[49] Corbu AR Rotaru A Nour V. Edible vegetable oils enriched with carotenoids extracted from by-products of sea buckthorn (*Hippophae rhamnoides* ssp. sinensis): the investigation of some characteristic properties, oxidative stability and the effect on thermal behaviour. *J Therm Anal Calorim.* (2020) 142:735–47. 10.1007/s10973-019-08875-5

[50] Huang J Guo X Xu T Fan L Zhou X Wu S. Ionic deep eutectic solvents for the extraction and separation of natural products. *J Chromatogr A.* (2019) 1598:1–19. 10.1016/j.chroma.2019.03.046 31005289

[51] Vinatoru M. Ultrasonically assisted extraction (UAE) of natural products some guidelines for good practice and reporting. *Ultrason Sonochem.* (2015) 25:94–5. 10.1016/j.ultsonch.2014.10.003 25454822

[52] Plazzotta S Ibarz R Manzocco L Martín-Belloso O. Optimizing the antioxidant biocompound recovery from peach waste extraction assisted by ultrasounds or microwaves. *Ultrason Sonochem.* (2020) 63:104954. 10.1016/j.ultsonch.2019.104954 31945560

[53] Gullón B Eibes G Moreira MT Herrera R Labidi J Gullón P. Yerba mate waste: a sustainable resource of antioxidant compounds. *Ind Crops Prod.* (2018) 113:398–405. 10.1016/j.indcrop.2018.01.064

[54] Ordóñez-Santos LE Esparza-Estrada J Vanegas-Mahecha P. Ultrasound-assisted extraction of total carotenoids from mandarin epicarp and application as natural colorant in bakery products. *LWT* (2021) 139:110598. 10.1016/j.lwt.2020.110598

[55] Goula AM Ververi M Adamopoulou A Kaderides K. Green ultrasound-assisted extraction of carotenoids from pomegranate wastes using vegetable oils. *Ultrason Sonochem.* (2017) 34:821–30. 10.1016/j.ultsonch.2016.07.022 27773309

[56] Meregalli MM Puton BMS Camera FDM Amaral AU Zeni J Cansian RL Conventional and ultrasound-assisted methods for extraction of bioactive compounds from red araçá peel (*Psidium cattleianum* Sabine). *Arab J Chem.* (2020) 13:5800–9. 10.1016/j.arabjc.2020.04.017

[57] Grillo G Gunjeviæ V Radoševiæ K Redovnikoviæ IR Cravotto G. Deep eutectic solvents and nonconventional technologies for blueberry-peel extraction: kinetics, anthocyanin stability, and antiproliferative activity. *Antioxidants.* (2020) 9:1069. 10.3390/antiox9111069 33142668 PMC7693902

[58] Liu Y Li J Fu R Zhang L Wang D Wang S. Enhanced extraction of natural pigments from Curcuma longa L. using natural deep eutectic solvents. *Ind Crops Prod.* (2019) 140:111620. 10.1016/j.indcrop.2019.111620

[59] Ozturk B Parkinson C Gonzalez-Miquel M. Extraction of polyphenolic antioxidants from orange peel waste using deep eutectic solvents. *Sep Purif Technol.* (2018) 206:1–13. 10.1016/j.seppur.2018.05.052

[60] Ferarsa S Zhang W Moulai-Mostefa N Ding L Jaffrin MY Grimi N. Recovery of anthocyanins and other phenolic compounds from purple eggplant peels and pulps using ultrasonic-assisted extraction. *Food Bioprod Process.* (2018) 109:19–28. 10.1016/j.fbp.2018.02.006

[61] Bosiljkov T Dujmiæ F Cvjetko Bubalo M Hribar J Vidrih R Brnèiæ M Natural deep eutectic solvents and ultrasound-assisted extraction: green approaches for extraction of wine lees anthocyanins. *Food Bioprod Process.* (2017) 102:195–203. 10.1016/j.fbp.2016.12.005

[62] Condurache NN Aprodu I Crãciunescu O Tatia R Horincar G Barbu V Probing the functionality of bioactives from eggplant peel extracts through extraction and microencapsulation in different polymers and whey protein hydrolysates. *Food Bioproc Technol.* (2019) 12:1316–29. 10.1007/s11947-019-02302-1

[63] Chuyen HV Nguyen MH Roach PD Golding JB Parks SE. Microwave-assisted extraction and ultrasound-assisted extraction for recovering carotenoids from Gac peel and their effects on antioxidant capacity of the extracts. *Food Sci Nutr.* (2018) 6:189–96. 10.1002/fsn3.546 29387378 PMC5778220

[64] Ramos M Jiménez A Garrigós MC. Il-based advanced techniques for the extraction of value-added compounds from natural sources and food by-products. *Trends Anal Chem.* (2019) 119:115616. 10.1016/j.trac.2019.07.027

[65] Martins N Ferreira ICFR. Wastes and by-products: upcoming sources of carotenoids for biotechnological purposes and health-related applications. *Trends Food Sci Technol.* (2017) 62:33–48. 10.1016/j.tifs.2017.01.014

[66] Gulzar S Raju N Chandragiri Nagarajarao R Benjakul S. Oil and pigments from shrimp processing by-products: extraction, composition, bioactivities and its application- A review. *Trends Food Sci Technol.* (2020) 100:307–19. 10.1016/j.tifs.2020.04.005

[67] Machado APDF Pereira ALD Barbero GF Martínez J. Recovery of anthocyanins from residues of Rubus fruticosus, Vaccinium myrtillus and Eugenia brasiliensis by ultrasound assisted extraction, pressurized liquid extraction and their combination. *Food Chem.* (2017) 231:1–10. 10.1016/j.foodchem.2017.03.060 28449984

[68] Laqui-Vilca C Aguilar-Tuesta S Mamani-Navarro W Montaño-Bustamante J Condezo-Hoyos L. Ultrasound-assisted optimal extraction and thermal stability of betalains from colored quinoa (*Chenopodium quinoa* Willd) hulls. *Ind Crops Prod.* (2018) 111:606–14. 10.1016/j.indcrop.2017.11.034

[69] Carrillo C Nieto G Martínez-Zamora L Ros G Kamiloglu S Munekata PES Novel approaches for the recovery of natural pigments with potential health effects. *J Agric Food Chem.* (2022). [Epub ahead of print]. 10.1021/acs.jafc.1c07208 35040324 PMC9204822

[70] Gómez-García MD Ochoa-Alejo N. Biochemistry and molecular biology of carotenoid biosynthesis in chili peppers (*Capsicum* spp.). *Int J Mol Sci.* (2013) 14:19025–53. 10.3390/ijms140919025 24065101 PMC3794819

[71] Delgado-Vargas F. *Natural Colorants for Food and Nutraceutical Uses.* Boca Raton, FL: CRC press (2002).

[72] Carvalho Gualberto N Santos de Oliveira C Pedreira Nogueira J Silva de Jesus M Caroline Santos Araujo H Rajan M Bioactive compounds and antioxidant activities in the agro-industrial residues of acerola (*Malpighia emarginata* L.), guava (*Psidium guajava* L.), genipap (*Genipa americana* L.) and umbu (*Spondias tuberosa* L.) fruits assisted by ultrasonic or shaker extraction. *Food Res Int.* (2021) 147:110538. 10.1016/j.foodres.2021.110538 34399515

[73] Umair M Jabbar S Nasiru MM Lu Z Zhang J Abid M Ultrasound-Assisted Extraction of Carotenoids from Carrot Pomace and Their Optimization through Response Surface Methodology. *Molecules.* (2021) 26:6763. 10.3390/molecules26226763 34833855 PMC8618288

[74] Humphrey AM. Chlorophyll as a color and functional ingredient. *J Food Sci.* (2004) 69(5):C422–5.

[75] Ngamwonglumlert L Devahastin S Chiewchan N. Natural colorants: pigment stability and extraction yield enhancement via utilization of appropriate pretreatment and extraction methods. *Crit Rev Food Sci Nutr.* (2017) 57:3243–59. 10.1080/10408398.2015.1109498 26517806

[76] Varo MA Jacotet-Navarro M Serratosa MP Mérida J Fabiano-Tixier AS Bily A Green ultrasound-assisted extraction of antioxidant phenolic compounds determined by high performance liquid chromatography from bilberry (*Vaccinium myrtillus* L.) juice by-products. *Waste Biomass Valori* (2019) 10:1945–55. 10.1007/s12649-018-0207-z

[77] Valavanidis A Vlachogianni T. Chapter 8 - Plant Polyphenols: recent advances in epidemiological research and other studies on cancer prevention. *Stud Nat Prod Chem.* (2013) 39:269–95. 10.5005/jp/books/10718_17

[78] Azeredo HMC. Betalains: properties, sources, applications, and stability – a review. *Int J Food Sci Technol.* (2009) 44:2365–76. 10.1111/j.1365-2621.2007.01668.x

[79] Gandía-Herrero F Escribano J García-Carmona F. Biological activities of plant pigments betalains. *Crit Rev Food Sci Nutr.* (2016) 56:937–45. 10.1080/10408398.2012.740103 25118005

[80] Rahimi P Mesbah-Namin SA Ostadrahimi A Separham A Asghari Jafarabadi M. Betalain- and betacyanin-rich supplements’ impacts on the PBMC SIRT1 and LOX1 genes expression and Sirtuin-1 protein levels in coronary artery disease patients: a pilot crossover clinical trial. *J Funct Foods.* (2019) 60:103401. 10.1016/j.jff.2019.06.003

[81] Tiwari S Upadhyay N Singh AK Meena GS Arora S. Organic solvent-free extraction of carotenoids from carrot bio-waste and its physico-chemical properties. *J Food Sci Technol.* (2019) 56:4678–87. 10.1007/s13197-019-03920-5 31686699 PMC6801288

[82] Benmeziane A Boulekbache-Makhlouf L Mapelli-Brahm P Khaled Khodja N Remini H Madani K Extraction of carotenoids from cantaloupe waste and determination of its mineral composition. *Food Res Int.* (2018) 111:391–8. 10.1016/j.foodres.2018.05.044 30007701

[83] Anticona M Blesa J Lopez-Malo D Frigola A Esteve MJ. Effects of ultrasound-assisted extraction on physicochemical properties, bioactive compounds, and antioxidant capacity for the valorization of hybrid Mandarin peels. *Food Biosci.* (2021) 42:101185. 10.1016/j.fbio.2021.101185

[84] Saini A Panesar PS. Beneficiation of food processing by-products through extraction of bioactive compounds using neoteric solvents. *LWT.* (2020) 134:110263. 10.1016/j.lwt.2020.110263

[85] Mercado-Mercado G Montalvo-González E Sánchez-Burgos JA Velázquez-Estrada RM Álvarez-Parrilla E González-Aguilar GA Optimization of β-carotene from ‘ataulfo’ mango (mangifera indica l.) By-products using ultrasound-assisted extraction. *Rev Mex Ing Quím.* (2019) 18:1051–61. 10.24275/uam/izt/dcbi/revmexingquim/2019v18n3/mercado

[86] Shahram H Dinani ST. Optimization of ultrasonic-assisted enzymatic extraction of β-carotene from orange processing waste. *J Food Proc Eng.* (2019) 42:e13042.

[87] Li N Li J Ding D Xie J Zhang J Li W Optimum parameters for extracting three kinds of carotenoids from pepper leaves by response surface methodology. *Separations.* (2021) 8:134. 10.3390/separations8090134

[88] Sharma M Bhat R. Extraction of carotenoids from pumpkin peel and pulp: comparison between innovative green extraction technologies (Ultrasonic and Microwave-Assisted Extractions Using Corn Oil). *Foods.* (2021) 10:787. 10.3390/foods10040787 33917570 PMC8067522

[89] Song J Yang Q Huang W Xiao Y Li D Liu C. Optimization of trans lutein from pumpkin (*Cucurbita moschata*) peel by ultrasound-assisted extraction. *Food Bioprod Process.* (2018) 107:104–12. 10.1016/j.fbp.2017.10.008

[90] Chutia H Mahanta CL. Green ultrasound and microwave extraction of carotenoids from passion fruit peel using vegetable oils as a solvent: optimization, comparison, kinetics, and thermodynamic studies. *Innov Food Sci Emerg Technol.* (2021) 67:102547. 10.1016/j.ifset.2020.102547

[91] Bhimjiyani VH Borugadda VB Naik S Dalai AK. Enrichment of flaxseed (*Linum usitatissimum*) oil with carotenoids of sea buckthorn pomace via ultrasound-assisted extraction technique: enrichment of flaxseed oil with sea buckthorn. *Curr Res Food Sci.* (2021) 4:478–88. 10.1016/j.crfs.2021.07.006 34382006 PMC8334381

[92] Sharma M Hussain S Shalima T Aav R Bhat R. Valorization of seabuckthorn pomace to obtain bioactive carotenoids: an innovative approach of using green extraction techniques (ultrasonic and microwave-assisted extractions) synergized with green solvents (edible oils). *Ind Crops Prod.* (2022) 175:114257. 10.1016/j.indcrop.2021.114257

[93] Mitrea L Cãlinoiu L-F Martãu G-A Szabo K Teleky B-E Mureşan V Poly(vinyl alcohol)-based biofilms plasticized with polyols and colored with pigments extracted from tomato by-products. *Polymers.* (2020) 12:532. 10.3390/polym12030532 32131384 PMC7182853

[94] Rahimi S Mikani M. Lycopene green ultrasound-assisted extraction using edible oil accompany with response surface methodology (RSM) optimization performance: application in tomato processing wastes. *Microchem J.* (2019) 146:1033–42. 10.1016/j.microc.2019.02.039

[95] Szabo K Emõke Teleky B Ranga F Simon E Lelia Pop O Babalau-Fuss V Bioaccessibility of microencapsulated carotenoids, recovered from tomato processing industrial by-products, using in vitro digestion model. *LWT.* (2021) 152:112285. 10.1016/j.lwt.2021.112285

[96] Hu J Lu W Lv M Wang Y Ding R Wang L. Extraction and purification of astaxanthin from shrimp shells and the effects of different treatments on its content. *Rev Bras Farmacogn.* (2019) 29:24–9. 10.1016/j.bjp.2018.11.004

[97] Sharayei P Azarpazhooh E Zomorodi S Einafshar S Ramaswamy HS. Optimization of ultrasonic-assisted extraction of astaxanthin from green tiger (*Penaeus semisulcatus*) shrimp shell. *Ultrason Sonochem.* (2021) 76:105666. 10.1016/j.ultsonch.2021.105666 34271396 PMC8283324

[98] Sinthusamran S Benjakul S Kijroongrojana K Prodpran T Agustini TW. Yield and chemical composition of lipids extracted from solid residues of protein hydrolysis of Pacific white shrimp cephalothorax using ultrasound-assisted extraction. *Food Biosci.* (2018) 26:169–76. 10.1016/j.fbio.2018.10.009

[99] Molina AK Gomes LC Palmeira L Pereira C Dias MI Ferreira IC *Extraction of Chlorophylls from Bioresidues of Daucus Carota L.(Carrots) Aerial Parts For Food Colorants Development. RETASTE: Rethink Food Waste*. Bragança: Instituto Politécnico de Bragança (2021).

[100] Chaiareekitwat S Latif S Mahayothee B Khuwijitjaru P Nagle M Amawan S Protein composition, chlorophyll, carotenoids, and cyanide content of cassava leaves (*Manihot esculenta* Crantz) as influenced by cultivar, plant age, and leaf position. *Food Chem.* (2022) 372:131173. 10.1016/j.foodchem.2021.131173 34601424

[101] Zulqarnain A Durrani AI Saleem H Rubab S. Development of an ultrasonic-assisted extraction technique for the extraction of natural coloring substance chlorophyll from leaves of *Carica papaya*. *J Oleo Sci.* (2021) 70:1367–72. 10.5650/jos.ess21118 34615827

[102] Kashaninejad M Sanz MT Blanco B Beltrán S Niknam SM. Freeze dried extract from olive leaves: valorisation, extraction kinetics and extract characterization. *Food Bioprod Process.* (2020) 124:196–207. 10.1016/j.fbp.2020.08.015

[103] Bruno Romanini E Misturini Rodrigues L Finger A Perez Cantuaria Chierrito T Regina da Silva Scapim M Ultrasound assisted extraction of bioactive compounds from BRS Violet grape pomace followed by alginate-Ca2+ encapsulation. *Food Chem.* (2021) 338:128101. 10.1016/j.foodchem.2020.128101 33091979

[104] Caldas TW Mazza KEL Teles ASC Mattos GN Brígida AIS Conte-Junior CA Phenolic compounds recovery from grape skin using conventional and non-conventional extraction methods. *Ind Crops Prod.* (2018) 111:86–91. 10.1016/j.indcrop.2017.10.012

[105] Dranca F Oroian M. Total monomeric anthocyanin, total phenolic content and antioxidant activity of extracts from eggplant (*Solanum Melongena* L.) peel using ultrasonic treatments. *J Food Process Eng.* (2017) 40:e12312. 10.1111/jfpe.12312

[106] Backes E Pereira C Barros L Prieto MA Genena AK Barreiro MF Recovery of bioactive anthocyanin pigments from *Ficus carica* L. peel by heat, microwave, and ultrasound based extraction techniques. *Food Res Int.* (2018) 113:197–209. 10.1016/j.foodres.2018.07.016 30195514

[107] Albuquerque BR Pinela J Barros L Oliveira MBPP Ferreira ICFR. Anthocyanin-rich extract of jabuticaba epicarp as a natural colorant: optimization of heat- and ultrasound-assisted extractions and application in a bakery product. *Food Chem.* (2020) 316:126364. 10.1016/j.foodchem.2020.126364 32058190

[108] Fernandes FAN Fonteles TV Rodrigues S de Brito ES Tiwari BK. Ultrasound-assisted extraction of anthocyanins and phenolics from jabuticaba (*Myrciaria cauliflora*) peel: kinetics and mathematical modeling. *J Food Sci Technol.* (2020) 57:2321–8. 10.1007/s13197-020-04270-3 32431358 PMC7230117

[109] Leichtweis MG Pereira C Prieto MA Barreiro MF Baraldi IJ Barros L Ultrasound as a rapid and low-cost extraction procedure to obtain anthocyanin-based colorants from *Prunus spinosa* L. Fruit Epicarp: comparative Study with Conventional Heat-Based Extraction. *Molecules.* (2019) 24:573. 10.3390/molecules24030573 30764526 PMC6384548

[110] More PR Arya SS. Intensification of bio-actives extraction from pomegranate peel using pulsed ultrasound: effect of factors, correlation, optimization and antioxidant bioactivities. *Ultrason Sonochem.* (2021) 72:105423. 10.1016/j.ultsonch.2020.105423 33383542 PMC7803825

[111] Chen L Yang M Mou H Kong Q. Ultrasound-assisted extraction and characterization of anthocyanins from purple corn bran. *J Food Process Preserv.* (2018) 42:e13377. 10.1111/jfpp.13377

[112] Xue H Tan J Li Q Tang J Cai X. Ultrasound-assisted enzymatic extraction of anthocyanins from raspberry wine residues: process optimization, isolation, purification, and bioactivity determination. *Food Anal Methods.* (2021) 14:1369–86. 10.1007/s12161-021-01976-8

[113] Milea AŞ Vasile AM Cîrciumaru A Dumitraşcu L Barbu V Râpeanu G Valorizations of sweet cherries skins phytochemicals by extraction, microencapsulation and development of value-added food products. *Foods.* (2019) 8:188. 10.3390/foods8060188 31159360 PMC6617110

[114] Chen X Wei Z Zhu L Yuan X Wei D Peng W Efficient approach for the extraction and identification of red pigment from *Zanthoxylum bungeanum* Maxim and Its Antioxidant Activity. *Molecules.* (2018) 23:1109. 10.3390/molecules23051109 29738434 PMC6100252

[115] Attaribo T Jiang X Huang G Zhang B Xin X Zhang Y Studies on the interactional characterization of preheated silkworm pupae protein (SPP) with anthocyanins (C3G) and their effect on anthocyanin stability. *Food Chem.* (2020) 326:126904. 10.1016/j.foodchem.2020.126904 32413765

[116] Chen X Guan Y Zeng M Wang Z Qin F Chen J Effect of whey protein isolate and phenolic copigments in the thermal stability of mulberry anthocyanin extract at an acidic pH. *Food Chem.* (2022) 377:132005. 10.1016/j.foodchem.2021.132005 34998152

[117] Guo Z Huang Y Huang J Li S Zhu Z Deng Q Formation of protein-anthocyanin complex induced by grape skin extracts interacting with wheat gliadins: multi-spectroscopy and molecular docking analysis. *Food Chem.* (2022) 385:132702. 10.1016/j.foodchem.2022.132702 35313199

[118] Šeremet D Durgo K Jokiæ S Huðek A Vojvodiæ Cebin A Mandura A Valorization of banana and red beetroot peels: determination of basic macrocomponent composition, application of novel extraction methodology and assessment of biological activity in vitro. *Sustainability.* (2020) 12:4539. 10.3390/su12114539

[119] Roriz CL Heleno SA Alves MJ Oliveira MBPP Pinela J Dias MI Red pitaya (*Hylocereus costaricensis*) peel as a source of valuable molecules: extraction optimization to recover natural colouring agents. *Food Chem.* (2022) 372:131344. 10.1016/j.foodchem.2021.131344 34818747

[120] Roriz CL Xavier V Heleno SA Pinela J Dias MI Calhelha RC Chemical and bioactive features of *Amaranthus caudatus* l. flowers and optimized ultrasound-assisted extraction of betalains. *Foods.* (2021) 10:779. 10.3390/foods10040779 33916443 PMC8067032

[121] Nutter J Fernandez MV Jagus RJ Agüero MV. Development of an aqueous ultrasound-assisted extraction process of bioactive compounds from beet leaves: a proposal for reducing losses and increasing biomass utilization. *J Sci Food Agric..* (2021) 101:1989–97. 10.1002/jsfa.10815 32914436

[122] Guimarães B Polachini TC Augusto PED Telis-Romero J. Ultrasound-assisted hydration of wheat grains at different temperatures and power applied: effect on acoustic field, water absorption and germination. *Chem Eng Process Process Intensificat.* (2020) 155:108045. 10.1016/j.cep.2020.108045

[123] PubChem. *Open chemistry database at the National Institutes of Health (NIH).* Bethesda MD: National Center for Biotechnology Information, National Library of Medicine (2022).

