# Supplementary material for: Ultrasound-Assisted Extraction of Natural Pigments From Food Processing By-Products: A Review
Source: Front Nutr. 2022 May 24;9:891462. doi: 10.3389/fnut.2022.891462 (PMC9171369; doi:10.3389/fnut.2022.891462)
Supplement: Supplementary file 1 [file Table_1.DOCX]

Supplementary Material

**Table 1.** Application of UAE to obtain carotenoids from by-products

| **Source** | **By-product type** | **Carotenoids identified** | **US device** | **UAE experimental conditions** | **Reference** |
| --- | --- | --- | --- | --- | --- |
| Acerola (*Malpighia emarginata* L.), Umbu (*Spondias tuberosa* L.) | Seeds and peels | β-carotene, Canthaxanthin, Lycopene | US bath (40kHz) | Sample: powder t = 30 min  T = 25 °C Solvent = acetone 80% S/L ratio = 200 g/L | (Carvalho Gualberto et al. 2021) |
| Carrot (*Daucus carota*) | Pomace | TCC | US probe (750 W) | Sample: blanched frozen pomace t = 2 - 12 min P = 750 W Solvent = flaxseed oil S/L ratio = 1000 g/L Optimum: 12 min at 750 W | (Tiwari et al. 2019) |
| Carrot (*Daucus carota*) | Pomace | β-carotene, lutein | US probe (750 W, 20 kHz) | Sample: freeze-dried powder t = 3 - 37 min  T = 10 - 60 °C  Solvent = ethanol 13-97% S/L ratio = 20 g/L Optimum: 16 min, 29 °C, ethanol 59% | (Umair et al. 2021) |
| Cantaloupe (*Cucumis melo* L.) | Peel | β-carotene, lutein | US probe (20 kHz) | Sample: powder t = 10 - 40 min  T = 21 °C  Solvent = Hexane/acetone; hexane/ethanol; hexane/acetone/ethanol S/L ratio = 16.6 - 33.3 g/L Optimum: Hexane/acetone (70:30); 10 min, A = 100%, 18 g/L | (Benmeziane et al. 2018) |
| Kinnow Mandarin (*Citrus reticulata*) | Peel | Lutein | US probe (20 kHz, 500 W) | Sample: powder t = 15 - 60 min  T = 20 - 60 °C  A = 20 - 40% Solvent = Methanolic solution of KOH (20%)  S/L ratio = 83.3 g/L - 333 g/L Optimum: 40 min, A = 30%, 45 °C, 133 g/L | (Saini and Panesar 2020) |
|  | Peel | TCC | US bath (42 kHz, 240 W) | Sample: powder t = 40 - 60 min  T = 40 - 60°C Solvent = Sunflower oil S/L ratio = 0.4 - 1.2 g/L Optimum: 60 min, 60 °C, 0.4 g/L | Ordoñez-Santos (Ordóñez-Santos, Esparza-Estrada, and Vanegas-Mahecha 2021), 2021 |
| Hybrids mandarin: ‘Clemenvilla’, ‘Ortanique’ and ‘Nadorcott’ | Peel | TCC | US probe (20 kHz, 400 W) | Sample: fresh samples  t = 5, 15 y 30 min  T= 40 °C  P= 400 W Solvent = ethanol 50%  S/L ratio = 100 g/L Optimum: Hybrid “Nadorcott”, 30 min | (Anticona et al. 2021) |
| Gac Fruit (*Momordica cochinchinensis* Spreng.) | Peel | TCC | US bath (43.2 kHz, 250 W) | Sample: powder t = 10 - 100 min  T = 20 °C  P = 150, 200, 250 W Solvent = Ethyl acetate S/L ratio = 12.5 g/L Optimum: 250 W, 60 min, 20 °C, 12.5 g/L | (Chuyen et al. 2018) |
| Mango (*Manguifera indica var.* Ataulfo) | Peel and Paste | β-carotene | US probe (24 kHz, 400 W) | Sample: powder t = 10, 20, 30 min  T = 5 °C  A = 30, 65, 100%  Pulse: 0.4, 0.6, 0.8 s on Solvent = Tetrahydrofuran (THF) and butylated hydroxytoluene (BHT) S/L ratio = 100 g/L Optimum: 30 min, 30%, 0.4 s | (Mercado-Mercado et al. 2019), |
| Orange (*Citrus cinensis* L.) | Peel | TCC | US probe (26 kHz, 200 W) | Sample: Peels without essential oil t = 5 - 35 min  T = 20 – 50 °C  UI = 52 - 208 W/cm^2^  Solvent = d-limonene, n-hexane S/L ratio = 100, 200 g/L Optimum: 20 °C, 208 W/cm^2^, 5 min | (Boukroufa, Boutekedjiret, and Chemat 2017) |
| Orange (*Citrus cinensis* L. Osbeck) | Peel | TCC | US probe (20 kHz, 200 W) | Sample: Freeze-dried powder t = 5 min  A = 80%  Solvent = Ionic liquid:ethanol (1:2)  S/L ratio = 333 g/L Optimum: 1-n-butyl-3-methylimidazoliumtetrafluoroborate ([BMIM][BF4]) | (Murador et al. 2019), |
| Orange (*Citrus cinensis* Osb.*)* | Peel | TCC | US probe (500 W) | Sample: Fresh samples t = 5, 17.5, 30 min  T = 40 °C  P = 100, 250, 400 W  Solvent = Ethanol (0, 25, 50%)  S/L ratio = 100 g/L Optimum: 400 W, 30 min. ethanol 50% | (Montero-Calderon et al. 2019) |
| Orange (*Citrus cinensis*) | Pomace | TCC | US probe (20 kHz, 500 W) | Sample: powder t = 10 - 120 min  T = 25 - 30 °C  Enzyme: Pectinase 0-5%  pH = 3.5 - 6  Solvent = Ethanol  S/L ratio = 100 g/L Optimum: 120 min, pectinase 1.56% | (Shahram and Dinani 2019) |
| Pepper (*Capsicum annuum* L.) | Leaves | Zeaxanthin, Lutein epoxide, violaxanthin | US bath for saponification and UAE | Sample: freeze-dried powder Saponification time = 10 - 50 min  Saponification Solution: KOH: methanol 10 -30%  T = 55 °C  US time = 20 - 60 min  US Solvent = Acetone-ethyl acetate (1:2 v/v)  S/L ratio = 83 – 250 g/L Optimum: Saponification time = 30 min, and KOH: methanol 20%; US time = 40 min, 125 g/L. | (Li et al. 2021) |
| Pomegranate (*Punica granatum*) | Peel | TCC | US probe (20 kHz, 130 W) | Sample: powder  A = 20 - 60%  t = 0 - 60 min  T = 20 - 60 °C  Solvent = Sunflower and soy oil  S/L ratio = 100 - 333 g/L  Optimum: 30 min, 51.5 °C, 100 g/L, A = 58.8%, sunflower oil. | (Goula et al. 2017) |
| Pumpkin (*Cucurbita maxima*) | Peel | TCC | US probe | Sample: freeze-dried powder  A = 20%  T = 22 - 25 °C  t = 45 min  Pulse: 10s/5s on/off  Solvent = Green: Corn oil, S/L ratio = 100 g/L; Conventional: hexane:Isopropanol (60:40 v/v)  , S/L ratio = 200 g/L.  Optimum: Green solvent | (Sharma and Bhat 2021) |
| Pumpkin (*Cucurbita moschata*) | Peel | TCC  Lutein | US bath (100 kHz, 200 W) | Sample: powder  F = 45 - 100 kHz  P = 150 - 210 W  t = 30 - 50 min  T = 20 °C  Solvent = Hexane-ethanol-acetone-toluene (10:6:7:7)  S/L ratio = 25 - 33.3 g/L  Optimum: 203 W, 30 min, S/L: 32.25 g/L | (Song et al. 2018) |
| Purple passion fruit (*Passiflora edulis)* | Peel | TCC | US probe (100 W) | Sample: freeze-dried powder  t = 10 - 50 min  T = 30 - 60 °C  Solvent = Sunflower and olive oil  S/L ratio = 100-300 g/L  Optimum: Olive oil = 35 min, 47 °C, 290 g/L; Sunflower oil = 50 min, 49 °C, 219 g/L. | (Chutia and Mahanta 2021) |
| Sea buckthorn (*Hippophae rhamnoides*) | Pomace | TCC  β-carotene, zeaxanthin | US bath (35 kHz, 480 W) | Sample: powder  t = 50 min  T = 20 °C  Solvent = Extra virgin Sunflower oil (EVS), extra virgin Olive oil (EVO) and refined sunflower oil (RS)  S/L ratio = 25, 50, 100 g/L  Optimum: EVO, S/L 100 g/L | (Corbu, Rotaru, and Nour 2020) |
|  |  | TCC | US probe (20 kHz, 160 W) | Sample: solar dried powder  t = 15 - 80 min  T = 20 °C  A = 50 - 100%  Solvent = Flaxseed oil  S/L ratio = 5 - 20 (weight basis)  Optimum: 47.5 min, feed ratio 20, A = 75% | (Bhimjiyani et al. 2021) |
|  |  | Lutein,  Lycopene,  β-carotene, | US probe (200 W) | Sample: freeze-dried powder  t = 30 min  Pulse = 0.66  A = 40%  Solvent = refined corn oil (RC) and Extra virgin olive oil (EVO)  S/L ratio = 100 g/L  Optimum: EVO | (Sharma et al. 2022) |
| shrimp (*Penaeus vannamei*) | Waste | Astaxanthin | US bath (40 kHz, 90 W) | Sample: powder  t = 30 - 120 min  P = 30, 50, 70, 90 W  T = Room temperature  Solvent = Phosphonium based ILs: n-butanol: water (0.05:0.25:0.70; 0.13:0.25:0.62; 0.22:0.25:0.53; 0.32:0.25:0.43; 0.50:0.25:0.25)  S/L ratio = 50 g/L  Optimum: 50W, 60 min, Solvent = 0.13:0.25:0.62 | (Gao et al. 2020) |
|  | Cephalothorax | Astaxanthin | US probe (20 kHz, 750 W) | Sample: freeze-dried powder  t = 10 min  Pulse: 30:30 s on/off  A = 60 - 100%  Solvent = n-hexane: isopropanol (1:1 v/v)  S/L ratio = 125 – 250 g/L  Optimum: A = 80% | (Sinthusamran et al. 2018) |
| shrimp (*green tiger, Penaeus semisulcatus*) | Shell | Astaxanthin | US probe (20 kHz) | Sample: milled shrimp shell  t = 5 - 15min  T = 25 - 45 °C  A = 20 - 100%  Solvent = petroleum ether: acetone: water (15:75:10)  S/L ratio = 250 g/L  Optimum: A = 23.6%, 13.9min, 26.3 °C | (Sharayei et al. 2021) |
| Shrimp (*Pleoticus muelleri, Pandalus borealis*) | Shell | Astaxanthin | US bath (40 kHz) | Sample: powder  t = 20 - 40 min  T = 40 - 60 °C  Solvent = ethanol  S/L ratio = 111 - 200 g/L  Optimum: 142 g/L, 50 °C, 20min | (Hu et al. 2019) |
| Tomato (*Solanum lycopersicum* L.*)* | Peel | TCC  Lycopene  β-carotene | US bath (45 kHz) | Sample: powder  t = 5 - 20 min  T = 20 - 40 °C  Solvent = acetone  S/L ratio = 33.3 g/L  Optimum: 5 min, 20 °C | (Tsvetko, Milena, and Donka 2017) |
|  |  | Lycopene  Lutein  β-carotene | US bath (37 kHz) | Sample: powder  t = 10 min  T = 40 °C  Solvent = methanol, ethyl acetate, petroleum ether (1:1:1)  S/L ratio = 100 g/L | (Szabo et al. 2021) |
|  | Seeds | Lycopene | US probe | Sample: powder  t = 1.59 - 18.41 min  T = 40 °C  I = 30 – 70 W/m^2^  Solvent = Green extraction: virgin sunflower oil; Conventional extraction: hexane, methanol, acetone (2:1:1)  S/L ratio= 131.8 - 368.2 g/L  Optimum: 70 W/m^2^, 10min, 200 g/L | (Rahimi and Mikani 2019) |
|  | Peel and seeds | Lutein  Lycopene  β-carotene | US bath (37 kHz, 140/340 W) | Sample: powder  t = 30 min  T = 30 °C  Solvent = ethanol 98%  S/L ratio = 200 g/L | (Mitrea et al. 2020) |

Where: TCC = Total Carotenoids Content; US = ultrasound; A = US amplitude; P = US powder; I = US power intensity; F = US frequency; t = processing time; T = temperature of processing.
