# Supplementary material for: Ultrasound-Assisted Extraction of Natural Pigments From Food Processing By-Products: A Review
Source: Front Nutr. 2022 May 24;9:891462. doi: 10.3389/fnut.2022.891462 (PMC9171369; doi:10.3389/fnut.2022.891462)
Supplement: Supplementary file 2 [file Table_2.DOCX]

Supplementary Material

**Table 2.** Application of UAE to obtain anthocyanins from by-products

| **Source** | **By-product type** | **Anthocyanins identified** | **US device** | **UAE experimental conditions** | **Reference** |
| --- | --- | --- | --- | --- | --- |
| Bilberry (*Vaccinium myrtillus* L.) | Press cake, juice by-product | Galactosides derivates, glucosides derivates, arabinosides derivates, acetylgalactosides derivates, total acetylglucosides, total anthocyanins | US probe (1 kW) | Sample: freeze-dried powder  t = 0 - 60 min  T = 20 - 40 °C  I = 4.4 - 16.7 W/cm^2^  Solvent = Distilled water  S/L = 5 g/L  Optimum: 15 min, 16.7 W/cm^2^ | (Varo et al. 2019) |
| Blackberry (*Rubus fritucosus*) | Bagasse (seeds and peel) and pulp precipitate | TMA | US probe (20 kHz, 1500 W) | Sample: freeze-dried powder  t = 15 min  Pulse = 2s on / 4s off  A = 91%  Solvent = Deionized water  S/L = 40 g/L | (Zafra-Rojas et al. 2020) |
| Blackberry (*Rubus fritucosus*), Blueberry (*Vaccinium myrtillus* L.), and Grumixama (*Eugenia brasillensis*) | Residues from the industrial processing of juices and pulp | cyanidin-3-O-galactoside; cyanidin-3-O-acetyl-hexoside; cyanidin-3-O-rutinoside; cyanidin-3-O-glucoside; peonidin-3-O-glucoside; malvidin-3-O-galactoside; malvidin-3-O-glucoside; malvidin-3-O-arabinoside | US bath (37 kHz, 580 W) | Sample: wet residues  t = 90 min  T = 80 °C  Solvent = Acidified water pH 2.0; ethanol:water 50% and 70% v/v  S/L = 44.44 g/L  Optimum: ethanol 70% for Grumixama, Blackberry and Blueberry | (Machado et al. 2017) |
| Blackthorn (*Prunus spinosa* L.) | Epicarp | TAC | US bath (40 kHz) | Sample: freeze-dried powder  T = 5 - 25 min  P = 100 – 400 W  Solvent = ethanol 0-100% acidified with citric acid pH 3.0  S/L = 50 g/L  Optimum: 5 ± 0.15min, 400 ± 32W, 47.98 ± 2.88% | (Leichtweis et al. 2019) |
| Blueberry (*Vaccinium myrtillus* L.) | Peel | TAC | US probe (20 kHz, 100W) | Sample: frozen and milled  t = 5 - 40 min  T = 40 °C  Solvent = NADES:  Choline chloride (ChCl):malic acid (MA) 1.5:1; ChCl:citric acid (CA) 2:1; ChCl: lactic acid (LA) 1:1; ChCl:glycerol (Gly) 1:2  S/L = 50 g/L  Optimum: ChCl:LA; 30 min | (Grillo et al. 2020) |
| BRS Violet (BRS Rubea × IAC 1398-21) grapes | Pomace (Seeds, stalks and peel) | Cyanidin-3-O-glucoside | US probe | Sample: powder  t = 2.5, 5 and 10 min  Pulse = 5s on / 5s off  T = 25, 40 and 55 °C  A= 20,30 and 40%  Solvent = water  S/L = 5 g/L  Optimum: 55°C, A=40%, 6 min | (Bruno Romanini et al. 2021) |
| Eggplant (*Solanum melongena)* | Peel | delphinidin-3-rutinoside-5-glucoside; delphinidin-3-glucoside; delphinidin-3-rutinoside; cyanidin-3-rutinoside; petunidin-3-rutinoside | US bath (40 kHz, 100 W) | Sample: powder  T = 40 °C  Solvent = 70% ethanol solution  S/L = 50 g/L | (Condurache et al. 2019) |
|  |  | TAC | US bath | Sample: powder  t = 10 - 60 min  T = 50, 60 and 70 °C  F = 0,25,45 kHz  Solvent = methanol, ethanol and 2-propanol  S/L = 100 g/L  Optimum: methanol, 50min, 50 °C, 45 kHz. | (Dranca and Oroian 2017) |
| Fig (*Ficus carica* L.) | Peel | delphinidin-3-rutinoside, delphinidin-3-rutinoside-5-glucoside, petunidin-3-rutinoside, malvidin-3-rutinoside-5-glucoside and cyanidin-3-rutinoside. | US probe (12 kHz, 400W) | Sample: powder  T = 10 - 30 min  Pulse = duty cycle 0.5s  A = 100%  Solvent = distilled water (pH = 7), water with 0.01 M HCl (pH = 2) and 0.01 M NaOH (pH = 12), and ethanol-water (50 and 75%, v/v).  S/L = 100 g/L  Optimum: 30 min, acidified water | (Ferarsa et al. 2018) |
| Fig (*Ficus carica* L.) | Peel | cyanidin 3-rutinoside | US probe | Sample: freeze-dried powder  T = 5 - 55 min  T = 30 - 35 °C  P = 100-400 W  Solvent = ethanol 0-100 %v/v, acidified with citric acid to pH 3.0.  S/L = 50 g/L  Optimum: 21 min, 310 W and 100% ethanol | (Backes et al. 2018) |
| Grape (*Vitis vinifera*) | Pomace, juice by-products | four anthocyanin-3-O-monoglucosides (delphinidin, petunidin, peonidin, malvidin), two acylated derivatives (malvidin- and peonidin-3-acetylmonoglucosides) and two coumaroyl derivatives (peonidin- and malvidin-3-(6-O-p-coumaroyl) monoglucosides) | US bath (40 kHz, 100W) | Sample: freeze-dried powder  t = 50 min  T = 65 °C  Solvent = Acidified aqueous ethanol (70% ethanol, 0.1% HCl v/v); NADES: Choline chloride (ChCl):Citric Acid (CA); ChCl: Malic Acid (MA); ChCl: Proline (PR); MA; PR:MA; Betaine (BE):MA; BE:CA; MA:Glucose(GLU):Glycerol(GLY); MA:GLU with 25% v/v water.  S/L = 30 g/L  Optimum: ChCL:CA | (Panić et al. 2019) |
|  |  | TAC | US probe | Sample: Freeze thawed  t = 5 - 15 min  T = without control reached 88 °C  P = 250 - 450 W  Solvent = water acidified with 2% w/v citric acid  S/L = 333 g/L  Optimum: 15 min | (da Rocha and Noreña 2020) |
|  |  | TAC | US bath (39 KHz) | Sample: fresh and freeze-dried  t = 5 - 50 min  T = 15 - 60 °C  P = 0 - 100 W  Solvent = 1:1 ethanol:water  S/L = 50 g/L  Optimum: 50 min, 100W, 30°C | (González et al. 2020) |
|  | Pomace from wine residues | Malvidin-3-O-glucoside;  Petunidin-3-O-glucoside;  Peonidin-3-O-glucoside;  Delfinidin-3-O-glucoside;  Cyanidin-3-O-glucoside | US probe (20 kHz) | Sample: powder  t = 2 - 10 min  T = 28 ± 3 °C  Pd = 1000 W/L  Solvent = ethanol 50%  S/L = 100 g/L  Optimum: 9 min | (Caldas et al. 2018) |
|  | Lees | anthocyanin-3-O-monoglucosides  anthocyanin-3-(6-O-p-coumaroyl) monoglucosides | US bath (37 kHz) | Sample: powder  t = 15 - 45 min  T= 35 °C  P = 190 - 380 W  Solvent = NADES: Choline cloride:Malic acid (ChCl:MA) with water content 10 - 50%  S/L = 100 g/L  Optimum: 30.6 min, 341.5 W, 35.4 water content of NADES | (Bosiljkov et al. 2017) |
| Jabuticaba (*Myrciaria cauliflora*) | Peel | Cyanidin-3-O-glucoside | US bath | Sample: powder  t = 10 - 40 min  F = 25 and 40 kHz  Pd = 50 and 60 W/L  Solvent = Acidified water at pH 1.5, 3.0 and 7.0  S/L = 100 g/L  Optimum: 25 kHz, 10 min, pH 1.5 | (Fernandes et al. 2020) |
|  |  | TAC | US probe (20 kHz) | Sample: frozen and freeze-dried powder  t = 5 - 25 min  T =30 – 35 °C  P = 100 – 500 W  Solvent = Ethanol solution 0-100% v/v  S/L = 50 g/L  Optimum: 9.1 min, 75.8 °C, 418.9W, 20.3% ethanol | (Albuquerque et al. 2020) |
|  |  | TAC | US probe (19 kHz) | Sample: powder  T = 3 min  T =25 °C  I= 1.1 - 13 W/cm^2^  Solvent = Water/Ethanol 0, 25, 50, 75 and 100 g/100g  S/L = 40 g/L  Optimum: 7.3 W/cm^2^, 50% W/E | (Gadioli Tarone et al. 2021) |
| Mulberry (*Morus alba*) | Wine residues | TAC | US bath (40 kHz) | Sample: powder  t = 60 - 120 min  T =40 – 60 °C  P = 200 - 400 W  Enzyme dosage = 0.15, 0.20, 0.25%  Solvent = Distilled water acidified to pH 3.5  S/L = 50 g/L  Optimum: 52 °C, 315 W, 0.22% enzyme, 94 min. | (Zhang et al. 2020) |
| Peach (*Prunus persica*) | Ground peach waste | Cyanidin-3-O-glucoside | US probe (24 kHz) | Sample: Frozen-thawed and dried  t = 20, 70 and 120 s  T = <80 °C  A = 25, 75, 125 µm  Solvent = ethanol: water 70:30  S/L = 0.7 g/g  Optimum: frozen-thawed dispersion at 28.75 µm and 120s | (Plazzotta et al. 2020) |
| Pomegranate (*Punica granatum* var. Bhagwa) | Peel | TAC | US probe (20 kHz) | Sample: powder  t = 1 - 10 min  T = Room T  Pulse = 0.10 - 0.90  P = 70 – 210 W  A = 10 - 100 %  Solvent = ethanol 50%  S/L = 20 - 100 g/L  Optimum: 60 g/L, 140W, 50%A, 10min. | (More and Arya 2021) |
| Purple corn (*Zea Mays* L.) | Husk | TAC | US bath | Sample: powder  Pulse: 4s on / 8s off, 0-130 times  T = 40 °C  P = 100 – 500 W  Solvent = 95% ethanol: 0.1 mol L-1 citric acid: distilled water = 4:1:3, v:v:v  S/L = 125 g/L  Optimum: 90 times, 400W | (Chen, Yang, et al. 2018) |
| Raspberry (*Rubus idaeus* L.) | Wine residues | Cyanidin-3-glucoside Cyanidin-3-rutinoside | US probe (20 kHz) | Sample: freeze-dried powder  t = 20 - 40 min  T= 40 – 50 °C  P= 200 – 400 W  Enzyme dosage= 0.1-0.2% pectinase  Solvent = 60% ethanol acidified pH 3.0  S/L = 33.3 g/L  Optimum: 43.94 °C, 290.9 W, 0.16% enzyme, 30 min. | (Xue et al. 2021) |
| Red araçá (*Psidium cattleianum* S.) | Peel | TAC | US bath (40 kHz, 154 W) | Sample: powder  t = 90 min  T= 40 °C  Solvent = 90% ethanol  S/L = 100 g/L | (Meregalli et al. 2020) |
| Sweet cherries (*Prunus avium* L.) | Peel | TAC | US bath (40 kHz, 100 W) | Sample: freeze-dried powder  t = 30 min  T= 40 ± 0.5 °C  Solvent = 70% ethanol  S/L = 125 g/L | (Milea et al. 2019) |
| Huajiao (*Zanthoxylum bungeanum maxim*) | Peel | Cyanidin-3-glucoside  Cyanidin-3-rutinoside | US bath (40 kHz, 300 W) | Sample: pre-treated with ethyl acetate an air-dried powder  t = 10 - 50 min  T = 15 – 35 °C  Solvent = ethanol, purified water, 60% ethanol, methanol, 0.1% v/v hydrocloric acid in ethanol, 0.1% v/v hydrocloric acid, 0.1% hydrocloric acid in 60% ethanol, and 0.1% v/v hydrocloric acid in methanol.  S/L ratio = 28.6-66.7 g/L  Optimum: 33.3 g/L, 40 min, 25 °C, | (Chen, Wei, et al. 2018) |

Where: TMA = total monomeric anthocyanins; TAC = total anthocyanins content; US = ultrasound; A = US amplitude; P = US powder; I = US power intensity; Pd = US power density; F = US frequency; t = processing time; T = temperature of processing.
