# Supplementary material for: Ultrasound-Assisted Extraction of Natural Pigments From Food Processing By-Products: A Review
Source: Front Nutr. 2022 May 24;9:891462. doi: 10.3389/fnut.2022.891462 (PMC9171369; doi:10.3389/fnut.2022.891462)
Supplement: Supplementary file 3 [file Table_3.DOCX]

Supplementary Material

**Table 3.** Application of UAE to obtain betalins from by-products

| **Source** | **By-product type** | **Betalains identified** | **US device** | **UAE experimental conditions** | **Reference** |
| --- | --- | --- | --- | --- | --- |
| Red pitahaya (*Hylocereus costaricensis*) | Peels (waste from pulp juice making industry) | Betanin, Isobetanin,  Phyllocactin, Isophyllocactin | NS | Sample: freeze-dried powder t = 1 - 50 min P = 5 - 500 W Solvent = water:ethanol (90:10, v/v)  S/L ratio = 5 g/L Optimum: 38 min at 487 W | (Roriz et al. 2022) |
| Prickly pear (*Opuntia engelmannii* cultivar (cv.)) | Peels (food processing waste) | Betacyanins | US bath (40 kHz) | Sample: freeze-dried powder t = 0.5 - 1.5 min T = 5 - 27.5 °C Solvent = methanol (0-100%) pH 7 S/L ratio = 5-35 g/L Optimum: 1.5 min, (S/L) 5 g/L, (metOH) 50%, 20 °C | (Melgar et al. 2019) |
| Amaranth (*Amaranthus caudatus* L.) | Flowers (Plant waste) | Betacyanins: amaranthine, isoamaranthine, betanin, isobetanin | NS | Sample: freeze-dried powder t = 1–45 min P= 5–500 W T = 25–35 °C Solvent = Distilled water S/L ratio = 50 g/L Optimum: 13.3 min at 500 W | (Roriz et al. 2021) |
| Quinoa (*Chenopodium quinoa* *Willd*). Common names: M1 = Red Pasankalla; M13 = Bright red Pasankalla | Husks of the seeds (food processing waste) | Betacyanins and betaxanthins | US probe (100 W, 30 kHz, A = 140 µm) | Sample: quinoa hulls containing betacyanins (M1) and betaxanthins (M13). M1: t = 2 - 20 s; A= 60 - 90 %; pulse = 0.2 - 0.6 s on. M13: t = 10 - 40 s; A= 50 - 90 %; pulse = 0.1 - 0.8 s on. Solvent = MilliQ ultrapure water S/L ratio = 1:100 w/v Optimum for M1: A= 70%; pulse = 0.6; t = 9.2 s Optimum for M13: A= 90%; pulse = 0.7; t = 40 s | (Laqui-Vilca et al. 2018) |
| Beetroot (*Beta Vulgaris*) | Leaves (plant waste) | Betacyanins and betaxanthins | US probe | Sample: fresh in small pieces t = 10, 16 min P = 35 - 100 W Solvent = water S/L ratio = 1:20 - 3:20 w/v Optimum: 90 W, 1:20 S/L, 16 min | (Nutter et al. 2021) |
| Beetroot (*Beta Vulgaris*) | Peel (food processing waste) | Betacyanins and betaxanthins | US bath (37 kHz) | Sample: powder t = 30, 60 min P = 200 W Solvent = distilled water S/L ratio = 1:20 w/v Optimum: 30 min | (Šeremet et al. 2020) |
| Beetroot (*Beta Vulgaris*) | Pomace (waste from juicing industries) | Betacyanins and betaxanthins | US bath (44 kHz) | Sample: powder t = 30 min P = 35 W T = 30 °C Solvent = water and 20, 30, 50% v/v ethanol or methanol S/L ratio = 1:25 w/v Optimum: Ethanol 30% | (Fernando et al. 2021) |

Where: NS = Not specified; US = ultrasound; A = US amplitude; P = US powder; I = US power intensity; Pd = US power density; F = US frequency; t = processing time; T = temperature of processing.
